# Modern Advances in Magnetic Materials of Wireless Power Transfer Systems: A Review and New Perspectives

**DOI:** 10.3390/nano12203662

**Published:** 2022-10-18

**Authors:** De’an Wang, Jiantao Zhang, Shumei Cui, Zhi Bie, Kai Song, Chunbo Zhu, Milyaev Igor Matveevich

**Affiliations:** 1 School of Electric Engineering and Automation, Harbin Institute of Technology, Harbin 150001, China; 2 Baikov Institute of Metallurgy and Material Science, Russian Academy of Sciences, Moscow 119991, Russia

**Keywords:** wireless power transfer, magnetic coupler, magnetic material, nanocrystalline

## Abstract

The magnetic coupling resonant wireless power transfer (MCR-WPT) system is considered to be the most promising wireless power transfer (WPT) method because of its considerable transmission power, high transmission efficiency, and acceptable transmission distance. For achieving magnetic concentration, magnetic cores made of magnetic materials are usually added to MCR-WPT systems to enhance the coupling performance. However, with the rapid progress of WPT technology, the traditional magnetic materials gradually become the bottleneck that restricts the system power density enhancement. In order to meet the electromagnetic characteristics requirements of WPT systems, high-performance Mn-Zn and Ni-Zn ferrites, amorphous, nanocrystalline, and metamaterials have been developed rapidly in recent years. This paper introduces an extensive review of the magnetic materials of WPT systems, concluding with the state-of-the-art WPT technology and the development and application of high-performance magnetic materials. In addition, this study offers an exclusive reference to researchers and engineers who are interested in learning about the technology and highlights critical issues to be addressed. Finally, the potential challenges and opportunities of WPT magnetic materials are presented, and the future development directions of the technology are foreseen and discussed.

## 1. Introduction

Wireless power transfer (WPT) technology has developed rapidly in recent years because of its unique advantages over traditional cable power supply methods [1,2,3,4,5]. It can significantly improve the reliability, convenience, and safety of the electric energy supply and solve the problems of sparks and maintenance difficulties caused by traditional plug-in power transmission modes [6,7,8]. WPT technology has broad application prospects in low-power scenarios such as mobile phones [9,10], wearable devices [11,12], implantable medical care [13,14,15], and smart home products [16,17], as well as high-power fields such as electric vehicles (EVs) [18,19,20,21,22,23], unmanned aerial vehicles (UAVs) [24,25], unmanned underwater vehicles (UUVs) [26,27,28], electric ships [29] and aerospace equipment [30,31]. There is no doubt that WPT technology eliminates the problem of insulation and wire wear due to contact friction and significantly improves the safety and reliability of charging systems.

Generally, the magnetic coupling resonant wireless power transfer (MCR-WPT) technology is considered the most promising WPT method for its major transmission power, high transmission efficiency, and long transmission distance [32,33,34]. The working principle of the MCR-WPT is to generate an alternating current from a high-frequency inverter power supply and pass it into the transmitting coil to generate an alternating high-frequency magnetic field in the space around the transmitting coil. The high-frequency magnetic field passes through the receiving coil and forms the high-frequency induction current. Then, the current through the secondary energy conversion links (rectifier and filter), provides stable electric energy to the load. MCR-WPT technology can be applied to power transmission of W ~ kW level and adapted to a wide range of applications. Since both the primary and secondary sides of the coupling coil use a tuning circuit to make it work in the resonance state of the same frequency, the energy exchange efficiency is very high. In addition, the electric vehicle wireless power transfer (EV-WPT) international standard SAE-J2954 [35] was proposed and adopted, which recommended using the magnetic coupling resonant method for wireless charging of EVs.

As an essential component of the MCR-WPT system for energy conversion and transmission, the magnetic coupler (MC) can be regarded as a non-contact loose coupling transformer composed of coil winding and magnetic core [36]. The coil winding is the key to realizing magnetic coupling, while the magnetic core made of soft magnetic materials is often overlooked. Generally, for a complete MC, including the primary and secondary sides, the coil winding realizes the construction of the space electromagnetic field based on Faraday’s electromagnetic induction law, and the magnetic material realizes the reshaping, restriction, and guidance of the space magnetic path. This is due to the remarkable permeability of magnetic materials compared with air medium, and most of the flux lines generated by coils will pass through the magnetic path with magnetic cores. Therefore, high-performance soft magnetic materials are recommended to be added to the magnetic coupler structure as a magnetic core, which significantly effects the improvement of coupling performance and electromagnetic shielding [37,38,39]. On the one hand, magnetic materials can effectively improve the quality factor and mutual inductance coupling coefficient, which plays a vital role in enhancing the system’s power level and transmission efficiency. On the other hand, magnetic materials can effectively decrease the electromagnetic leakage of magnetic couplers and reduce the electromagnetic radiation to electronic equipment of the system and surrounding environment, which is conducive to the realization of the electromagnetic compatibility (EMC) design.

However, from a negative perspective, WPT systems generally work in the higher frequency region of magnetic materials, bringing additional power loss. In addition, the use of magnetic cores increases the volume, weight, and cost of the system. WPT is a new field of magnetic materials with special needs and concerns different from the traditional application scenarios of magnetic materials (such as transformers and motors). As far as the research progress of magnetic materials for WPT is concerned, it is still in the stage of how to make good use of basic soft magnetic materials. Few magnetic material companies have proposed special magnetic materials specially designed and developed for WPT.

Nevertheless, with the deepening of the research on WPT technology, the contradiction between the requirements of high efficiency, high power density, low cost, lightweight, and the shortcomings of current WPT magnetic materials have gradually been exposed. The existing traditional magnetic materials will be challenging to adapt to the high-transmission performance requirements of the WPT system and may become a bottleneck restricting the further development of WPT technology. In brief, from the perspective of the current development heat of WPT technology in the field of consumer electronics and the promotion of EVs, the research on magnetic materials and magnetic structures of magnetic couplers in WPT systems will become a research hotspot in the future. It is also one of the critical ways to further break through the bottleneck problems mentioned above.

This paper introduces an extensive and focused review for magnetic materials in WPT technology, including the following:

– Section 2 presents the brief history, principles, and latest progress of MCR-WPT.
– Section 3 presents the development process of high-performance magnetic materials and their application in WPT technology.
– Critical issues and hotspots of WPT magnetic materials are presented in Section 4, while the potential opportunities and trends are summarized in Section 5.
– The outlook for magnetic materials and WPT technology is briefly described in Section 6, then the paper is concluded in Section 7.

## 2. History, Theory and Applications of WPT

### 2.1. Brief History of WPT

The turning point of the first realization of electric energy transmission from wire to wireless can be traced back to the last century: Hertz provided conclusive evidence of the existence of electromagnetic waves, and Nicola Tesla successfully lit the phosphorescent lamp with his Tesla coil [40,41,42]. However, embodiments thereof involve an unnecessarily large electric field. Until 1964, the microwave radiation type wireless charging system was first proposed and applied to supplement energy for a special helicopter [43]. Although microwave radiation is very suitable for transmitting information, it will cause a lot of energy loss due to the divergent radiation space when transmitting power, and the power transmission efficiency is very low. Since then, electromagnetic induction radio energy transmission based on Faraday’s electromagnetic induction law has become a new research direction [44,45,46,47,48] and has continued into the 21st century. On this basis, the research institute has made many research achievements in lightweight, high-power capacity and density, and high misalignment tolerance WPT systems [49,50,51,52,53,54,55,56].

It is difficult not to highlight some of these landmark achievements. In 1976, the concept of dynamic radio energy transmission was first introduced [57], and LBNL evaluated its system feasibility. Dynamic wireless power transfer (DWPT) realizes the energy supply during the operation of EVs and the unlimited mileage endurance of EVs under ideal conditions. In 2007, Marin Soljačić [58] from Massachusetts Institute of Technology (MIT) proposed a mid-range MCR-WPT system, which acted as a leading role in academia. In 2009, Korea Advanced Institute of Science and Technology (KAIST) developed the first on-line electric vehicle (OLEV) prototype [59]. In the following years, KAIST rapidly commercialized and updated its OLEV products [60,61,62,63], which was named as one of the top 50 inventions in the world by Time magazine.

### 2.2. Classification, Principle, and Comparison of WPT

**Figure 1:**
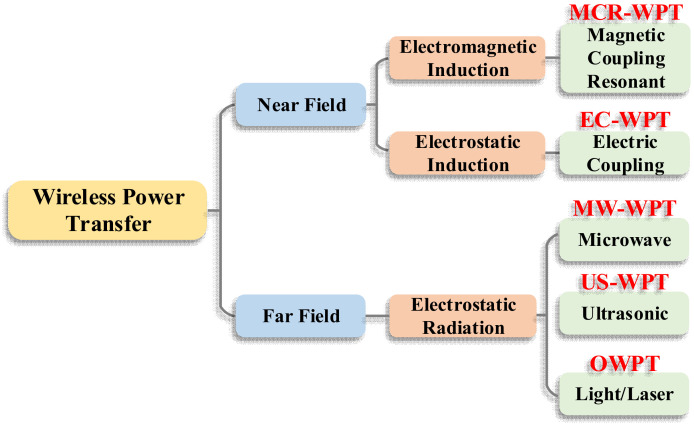
Classification of wireless power transfer technologies.

**Figure 2:**
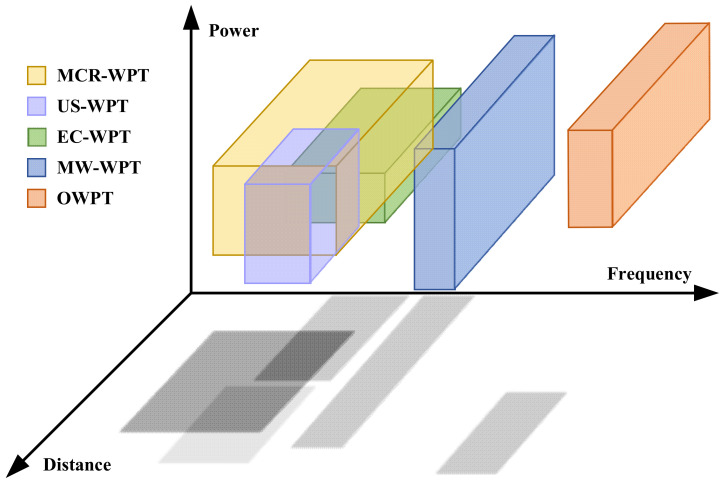
The performance of different types of wireless power transfer technologies in terms of power, frequency and distance.

The WPT technology can be realized based on different physical principles. The development of WPT technology is advancing in two major directions, near-field and far-field transmission, based on the distance of power transfer. Then, according to the power accumulation medium and transfer technique, the WPT technology is mainly divided into three types: electromagnetic induction type, electrostatic induction type, and electromagnetic radiation type [64,65,66]. The classification of WPT is shown in Figure 1. Moreover, WPT technology can be further divided into five categories: magnetic coupling resonant WPT (MCR-WPT), electric coupling WPT (EC-WPT), microwave WPT (MW-WPT), ultrasonic wireless power transfer (US-WPT), and optical wireless power transfer (OWPT). The operating frequency range, power range and applicable transmission distance of several WPT types can be visualized and specifically obtained from Figure 2 and Table 1.

#### 2.2.1. US-WPT and MW-WPT

**Figure 3:**
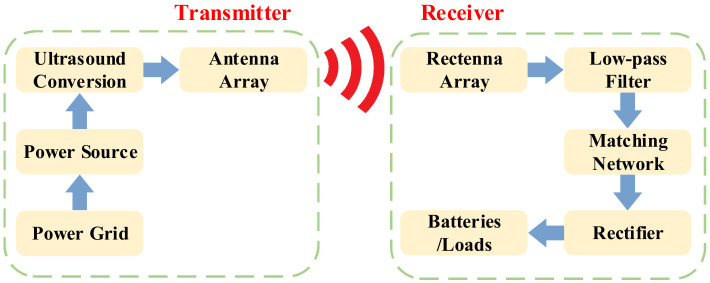
Components and fundamentals of the US-WPT system.

The US-WPT system transmits energy through space ultrasound radiation, and its principle is shown in Figure 3. The power amplifier converts the DC signal into ultrasounds, and the transmitting antenna then emits the ultrasound beam. The receiving antenna is used to receive the ultrasounds and eventually rectify them into a DC power supply that can be used by the device [67,68,69]. The ultrasonic transmission was originally designed to transmit information, so its power capacity is often low, usually not exceeding 10 W.

The transmission principle of MW-WPT is similar to that of US-WPT. Still, the frequency range of its band and the energy capacity are significantly different, while the MW-WPT can even realize the wireless transmission of MW-level energy. MW-WPT and US-WPT transmissions are radiant, so a large portion of the power is dissipated by radiation during transmission and cannot be captured by the receiving side, resulting in extremely low transmission efficiency [70]. With the improved antenna technology, directional antennas can already achieve directional MW-WPT [71]. However, directional MW-WPT is very sensitive to the transmission medium, does not allow any obstacles in its transmission path, and requires real-time tracking and positioning of microwaves, which is difficult to implement.

#### 2.2.2. OWPT

**Figure 4:**
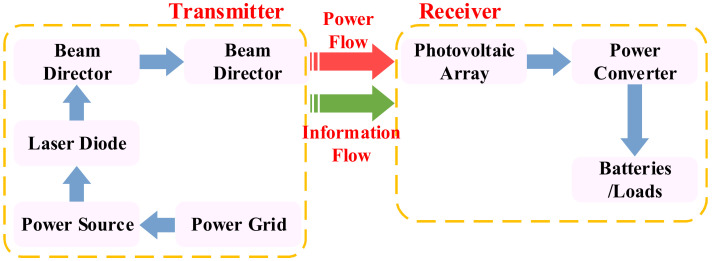
Components and fundamentals of the OWPT system.

Optical Wireless Power Transfer (OWPT) is one of the WPT technologies specifically researched for long-distance directional devices. The components and principles of the OWPT system are shown in Figure 4. The most significant advantage of OWPT is that it can realize long-distance and high-power directional energy transmission, and its transmission distance can even be greater than 1 km. At this stage, the leading service objects of OWPT include UAVs, satellites, and other remote power facilities.

The system composition and fundamental principle of OWPT are shown in Figure 4. The electricity in the power grid is converted into light via laser diodes. Then, the laser beam is shaped by optical elements and directed to a remote photovoltaic receiver through the beam director. The particular photovoltaic receiver can match the laser wavelength and beam intensity and convert the laser back to electric energy to charge the battery load [72,73,74]. The OWPT system is more like an ultralong transmission wire. However, its efficiency is very low, and there are high requirements for directivity and unshielded laser beams.

#### 2.2.3. EC-WPT

**Figure 5:**
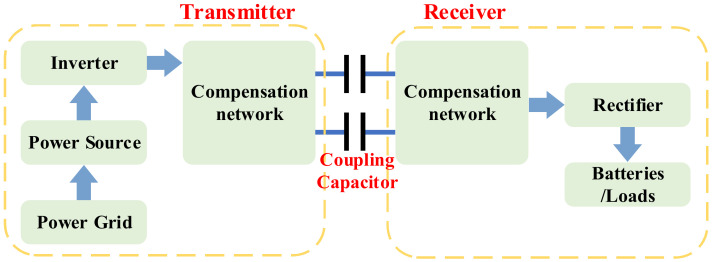
Components and fundamentals of the EC-WPT system.

The EC-WPT is usually referred to as capacitive power transfer (CPT), which usually uses two pairs of metal plates form an equivalent capacitor to transmit power. The system composition and basic principle of EC-WPT are shown in Figure 5. Due to its unique operating principle, EC-WPT can be used in applications requiring power transfer through metal materials [75]. Unlike the MCR-WPT system, the EC-WPT system transfers energy by the electric field, so it is unnecessary to carry a large number of magnetic cores. Thus, the EC-WPT system has a simple structure with no magnetic hysteresis loss. Furthermore, the main advantage of EC is that almost no electric flux can escape beyond the dielectric material, thus eliminating the problem of electromagnetic field exposure [76]. Due to the limitation of high resonant voltage, EC-WPT is usually designed as a system with high operating frequency and low power level [77,78].

With the continuous progress of technology, some kilowatt power level EC-WPT systems have been proposed, which can realize the wireless transfer of electric energy over a long distance [79,80,81]. In addition, the coupling capacitors are diversified in design for different applications. However, to improve the power level, it is necessary to increase the operating frequency of the system to more than MHz. The existing semiconductor technology limits the ultra-high switching frequency and high-power performance. Furthermore, continuous operation in a high-frequency range easily causes high-voltage stress on electronic devices in the compensation network. Gallium nitride (GaN) switching technology and multiphase modular design will be important solutions to overcome the development bottleneck of high-power EC-WPT systems [82].

#### 2.2.4. MCR-WPT

**Figure 6:**
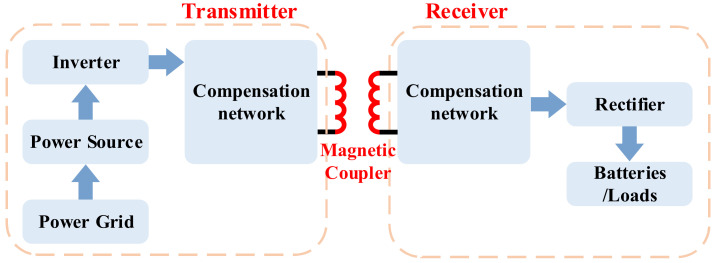
Components and fundamentals of the MCR-WPT system.

MCR-WPT is now the most widely accepted and applied WPT method. In the MC-WPT system, the receiving coil picks up the flux lines of the magnetic field generated by the high-frequency current in the transmitting coil and converts it into a DC current for charging. The components and fundamentals of MCR-WPT are shown in Figure 6. The magnetic coupler can be regarded as a loosely coupled transformer with a long distance between the primary and secondary windings [83]. The loosely coupled transformer commonly has a large airgap between the double side windings, which leads to a lower coupling coefficient and higher electromagnetic leakage. Through the utilization of the magnetic core, the quality factor of coupling coils can be optimized, and then the effective power transfer can be realized [84].

With the continuous development of MCR-WPT technology, the power level of the existing research has reached hundreds of kilowatts, and the transmission efficiency is very considerable. In addition, the multi-module parallel connection method can even realize megawatts of wireless power transfer [85]. The distance diameter ratio (DDR) is one of the key research directions of WPT systems. DDR is the ratio of the distance between the primary and secondary coils to the coil diameter. It is one of the parameters characterizing the transmission capacity of the magnetic coupler. In some long-distance MCR-WPT systems, the DDR can reach one or even higher. The misalignment tolerance of the magnetic coupler is another important characterization parameter. Through the optimization of magnetic coupler and compensation topology of MCR-WPT, the high misalignment tolerance characteristics can be realized. Such optimization can improve the flexibility and freedom of the MCR-WPT, so that the magnetic coupler can still achieve an efficient state when misalignment. The vertical and horizontal allowable misalignment tolerance can reach a level comparable to half of the coil diameter.

The main advantages and potential shortcomings of the five types of WPT techniques are detailed in Table 2.

### 2.3. The State-of-the-Art of MCR-WPT

**Figure 7:**
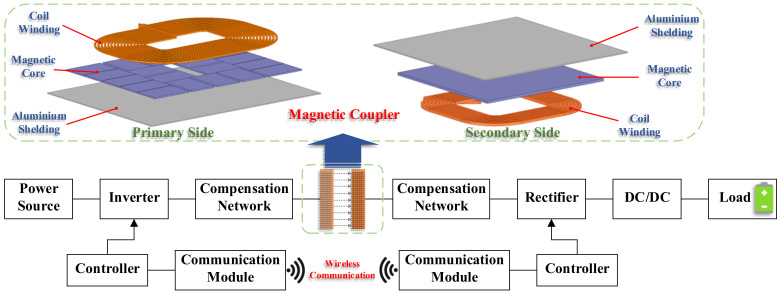
Composition of the MCR–WPT system and its magnetic coupler.

The structure of the MCR-WPT system is shown in Figure 7, including the inverter, the compensation network, the magnetic coupler, the rectifier, DC/DC module, and the load [86]. The significant difference between the magnetic coupling resonance type and the traditional inductive power transfer is that the MCR-WPT system adds a resonance compensation network to eliminate the reactive power component in the circuit, which means that the magnetic coupling resonant type can obtain higher active power transmission under the same degree of coil coupling. In other words, when in the case of a longer distance (weak coupling state), it still can have a higher active power output.

In recent years, many institutes have conducted in-depth research on WPT technology, mainly focusing on system modeling and control, magnetic coupler and compensation topology design, anti-misalignment capability, electromagnetic leakage, and shielding. The representative institutions include Auckland University [87], Korea Advanced Institute of Science and Technology (KAIST) [63,88,89,90], Korea Railroad Research Institute [85], Oak Ridge National Laboratory (ORNL) [91], University of Michigan [92], Saitama University [93], Harbin Institute of Technology (HIT) [94,95] and Chongqing University [96]. Table 3 shows some typical research results and related technical parameters of universities and research institutions.

In addition, companies such as Qualcomm Halo [97], WiTricity [98,99], Momentum Dynamics [100,101], Bombardier [102], ZTE New Energy, Zone Charge, and INVIS Power [103] are sparing no effort to promote the research of wireless power transfer technology and its application. The main electrical parameters of typical WPT products of the typical companies are shown in Table 4.

At present, hundreds of kilowatts power level and more than 90% transmission efficiency have been achieved in the existing research reports. However, compared with the conventional wired charging method, there are still problems of lower power density. At this stage, high efficiency and high-power density are the important development directions of the MCR-WPT technology.

With the continuous progress of power electronics technology, the new generation of SiC and GaN switching power electronics further reduces the switching losses of the inverters and rectifiers. Improving the Litz wire preparation process reduces the proximity effect and skin effect of the coil so that the copper loss of the magnetic coupler is further optimized. Therefore, improving the electromagnetic properties of magnetic materials has become one of the most effective solutions to break the bottleneck problem of increasing the power density of the WPT system. The development of soft magnetic materials with high permeability, high saturation magnetic induction, and low power consumption has gradually become the key to improving the efficiency of the WPT system.

## 3. Magnetic Materials and Their Applications in WPT

### 3.1. Brief History of Soft Magnetic Materials

In 1831, through an experiment, Michael Faraday found that when a part of the conductor of a closed circuit cut the magnetic induction line in the magnetic field, the current would be generated in the conductor. Faraday’s law of induction was proposed [104]. Iron is chosen as the magnetic core because of its highest saturation magnetization among all elements. In addition, it also has the characteristics of high permeability and low coercivity. Since then, soft magnetic materials have been developing continuously. Later, the researchers found that the annealing process of iron can improve its mechanical properties and reduce its coercivity through stress relief, making it more suitable for induction applications.

In 1900, British metallurgist Robert Hudfield invented non-oriented silicon steel by adding 3% silicon to iron, which improved both the resistivity and saturation magnetization [105]. In 1933, American metallurgist Norman Goss invented grain-oriented silicon steel by promoting grain growth along the direction of low anisotropy crystallization, thus further improving the saturation magnetization. Even today, due to the high saturation magnetization and relatively low cost of silicon steel, it still occupies the main share of the global soft magnet market. The most common applications of silicon steel are large transformers (oriented silicon steel) and motors (isotropic non-oriented silicon steel). However, low resistivity (~0.5 mΩ·m) makes silicon steel lose more at high frequency [106]. Recently, electrical steel manufacturers have developed a method to increase the silicon content in steel to 6.5% by using a chemical vapor deposition process [107]. This method can increase the resistivity of silicon steel material to 82 μΩ·cm but still cannot meet the current high-efficiency requirements of high-frequency power electronic equipment and high-speed motors.

In the 1910s, Gustav Elmen of Bell Laboratories carried out experiments on nickel-iron and discovered the nickel-rich (78%) permalloy composition [108]. A significant advantage of permalloy is its high relative permeability (up to 100,000). Nickel-iron is still used in some special induction applications today, but it is not common in power electronics and motors because of their high eddy current loss. Adding nickel can reduce soft magnetic materials’ saturated magnetic flux density. Moly permalloy powder (MPP) can be produced by adding a small amount of molybdenum (2%) to permalloy [109]. MPP is used to fabricate the powder cores with the lowest loss [110], and it is still the best choice for high-frequency inductor cores within the frequency range of 450 kHz.

Then in the late 1940s, the soft magnetic ferrites were invented by J. L. Snoek [111]. These materials have high resistivity, which can effectively suppress eddy current loss. In addition, the preparation process of ferrite is often simple so that the ferrite core can be produced at a very low cost. Soft ferrite has been developed rapidly in recent years due to its high resistivity and economic performance and has been widely used in electromagnetic induction and high-frequency equipment. Today, the market share of ferrite in the world’s soft magnetic materials is only second to that of silicon steel sheets [112]. Manganese zinc ferrite is also the most commonly used soft magnetic material in the WPT system at present. However, the saturation flux density of ferrite is relatively low (almost a quarter of that of silicon steel sheets), which limits the energy density of sensing elements containing ferrite cores. Therefore, increasing the maximum saturation flux density of soft ferrite has always been the development direction of the ferrite process.

In 1967, Duwez and Lin reported the first amorphous soft magnetic alloy in the form of small disc-shaped samples [113]. They used a rapid solidification technique called splat cooling for Fe-P-C systems. Then interest in Fe- and Co-based amorphous alloys surged by the mid of 1970s. Amorphous alloys obtained some applications because of their excellent coercivity and saturation magnetic density compared with ferrite. In 1988, Hitachi researchers added Nb and Cu additives and added an annealing step in the production of amorphous alloys to produce small and closely distributed the iron or cobalt-based nanocrystals (about 10 nm in diameter) in the matrix of amorphous materials, which marked the invention of nanocrystalline alloys [114]. Amorphous and nanocrystalline alloys have low power loss and competitive saturation flux density. Although the cost is higher than that of silicon steel, due to the low power loss, these advanced alloys can reduce the total lifetime cost of power electronics and motors.

In the early 1990s, powder cores (also known as soft magnetic composites or SMCs) were proposed [115]. These materials combine magnetic particles, anywhere between ~1 to 500 mm in diameter, and either coat or mix them with an insulating material before consolidating with high pressures. Moreover, the heat process can also be applied either during or after densification to improve magnetic properties. Magnetic particles are usually iron powder but can also be composed of alloys. Powder magnetic core can be quickly processed into a more complex magnetic core shape, improving its applicability in special equipment and significantly reducing manufacturing costs. Their isotropy, low cost, and the ability to make complex mesh parts make SMC quite successful in rotating electrical machines [116,117]. Although the magnetic permeability of the powder core is usually low, its stability at high frequencies is impressive (such as the MPP mentioned earlier). SMC-based magnetic cores are attractive in high-frequency inductor design. The desired overall core permeability of SMC core can be achieved by adjusting the powder size, addition of insulation material and phosphoric acid, and pressure during the preparation process to reduce the air gap loss and ease the inductor design [118].

**Figure 8:**
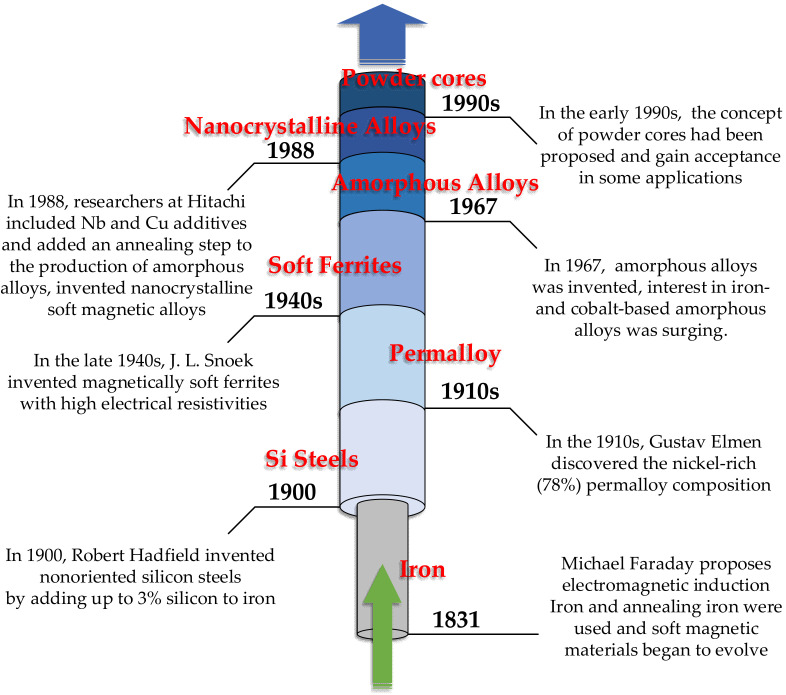
Research progress and a brief history of soft magnetic materials.

Figure 8 shows the brief history and development trend of soft magnetic materials [119,120]. From the perspective of power electronics applications, the saturated magnetic flux density, resistivity, and cost of soft magnetic materials are important concerns in their development. Ferrite material is a competitive core material at high frequency, so it has been in a leading position in the field of WPT technology. In recent years, with the application and popularization of advanced alloys such as amorphous and nanocrystalline alloys, researchers have gradually applied them to various WPT scenarios and obtained some applied results. In the following section, the application of soft magnetic materials and their magnetic structures in WPT technology will be discussed in detail.

### 3.2. Mn-Zn and Ni-Zn Soft Ferrites

Soft ferrite materials are the most widely used magnetic material in WPT systems at present and are also typical magnetic core materials recommended in SAE-J2954 and Qi standards [35,121,122]. They have remarkable performance in the field of consumer electronics and EVs wireless charging. Among them, Mn-Zn ferrites have high saturated magnetic flux density, permeability, and low resistivity compared with Ni-Zn ferrite. In addition, the performance of Mn-Zn ferrites is generally better than Ni-Zn below 2 MHz. The application of Mn-Zn ferrite materials accounts for about 80% of all ferrite materials.

According to different application conditions and performance indicators, Mn-Zn ferrite materials can be divided into two categories. One is high permeability ferrite (generally greater than 15,000), which is usually used in low-frequency broadband transformers and inductance components of communication equipment. The other is high-frequency low-loss ferrite, which is often called power ferrite. Under high frequency and magnetic flux density, the loss of power ferrites does not change much with the increase of temperature in a particular range [123,124]. Power ferrite materials can be used for power conversion and transmission due to the high magnetic flux density *B_s_*, high initial permeability *μ_i_*, and low power loss *P_c_* characteristics. Mn-Zn power ferrite materials were first used in household appliances, switching power supplies, and adaptive transformers. With the rise of WPT technology, Mn-Zn power ferrites have gradually become the most widely used core material.

At the end of the 20th century, material companies developed a set of landmark Mn-Zn power ferrite, which solved the problem of large power consumption and the rapid decline of magnetic properties at 100~500 kHz high-frequency ranges. The maximum saturated magnetic flux density of this high-performance power ferrite is about 500 mT, and the loss at high temperature and frequency (100 kHz, 200 mT, 100 °C) is about 400~500 kW/m^3^. The most well-known products include PC40 from TDK [125], 6H20 from FDK [126], and N72 from SIEMENS [127].

In the past decade, low *P_c_* ferrite materials have made remarkable progress. The PC45, PC46, and PC47 series Mn-Zn ferrites with low power loss near a temperature range are produced by TDK. The power consumption valley of PC45 is 60~80 °C, PC46 is 40~50 °C, and PC44 and PC47 are around 100 °C. Then, the PC90 and PC95 series Mn-Zn ferrite materials with wide temperatures and low power loss were developed. PC95′s *P_c_* was lower than 350 mW/cm^3^ in the range of 25~120 °C, while PC90′s *P_c_* was about 320 mW/cm^3^ at 100 °C. In addition, the *B_s_* of PC90 are 450 mT and 540 mT at 100 °C and 25 °C, respectively. The PC90 and PC95 series Mn-Zn ferrites have wide temperatures and low power loss with excellent comprehensive performance [128]. The performance parameters of TDK PC series Mn-Zn ferrites are shown in Table 5.

It is worth mentioning that the Netherlands’ Ferroxcube Company developed 3C series power ferrite materials [129]. 3C92 has high saturation magnetic induction, 3C95 has excellent temperature stability, and 3C94 has a low cost. 3C series Mn-Zn ferrite has comparable performance to TDK products, and the performance parameters of 3C ferrite materials are shown in Table 6.

In fact, the saturated magnetic flux density *B_s_* of Mn-Zn soft ferrite can theoretically reach 600 mT [130]. However, the products prepared by the prior art are lower than 500 mT no matter at room temperature 25 °C or high temperature. For the 4H45 material of the FDK Company, the *B_s_* is 450 mT at 100 °C, and the *B_s_* of 4H47 material is about 470 mT. The power consumptions of the two materials at 100 °C are 450 mW/cm^3^ and 650 mW/cm^3^, respectively [126]. Therefore, to this day, improving the saturated magnetic flux density *B_s_* of materials is still the research hotspot of Mn-Zn ferrite [131,132,133,134].

Compared with Mn-Zn ferrites, Ni-Zn ferrite materials are not very popular. Ni-Zn ferrites usually have low *B_s_* and *μ_i_*, but their resistivity is high, so it is more suitable for high-frequency applications. NiCuZn ferrite can be prepared by adding CuO to the main formula [135,136,137]. Because of its high resistivity, high-frequency characteristics, and low sintering temperature, it can be used to prepare laminated chip inductors [138]. The FDK Company proposed L47H [126] type Ni-Zn ferrite material for high-voltage power conversion, and the University of Electronic Science and Technology of China developed a similar HN120B type material [139].

As excellent high-frequency magnetic materials, MnZn ferrite and NiZn ferrite have been widely used in the field of wireless charging and mentioned in the two authoritative standards of Qi and SAE-J2954 [35,121]. Among them, the Qi standard proposed by the Wireless Power Consortium (WPC) is mainly for mobile consumer electronic devices such as mobile phones with the specified operating frequency range of 110~205 kHz. According to the Qi standard, application scenarios can be divided into fixed position type, single-coil free position type and multi-coil free position type. As the power level and current level of the products corresponding to the Qi standard are not high, the requirements for the temperature characteristics and saturated magnetic flux density of the required soft ferrite products are not strict. Table 7 shows the requirements for magnetic materials and corresponding recommended soft ferrite materials under the three application scenarios of the Qi standard [140,141,142,143,144].

SAE-J2954 standard is an international standard for the wireless charging of electric vehicles proposed by the Society of Automotive Engineers International (SAE International). SAE-J2954 imposed design constraints on three power levels (WPT1-3.7 kW, WPT2-7 kW, and WPT3-11 kW) [35]. The power capacity of EVs is much larger than mobile phones, so the magnetic field intensity generated by the magnetic coupler is significant [20,21]. In order to avoid the magnetic saturation failure of the magnetic core, the soft ferrite must have a high saturation flux density. In addition, the EV-WPT system will produce an obvious temperature rise during continuous operation, so there is an increased requirement for the temperature characteristics of soft ferrite. The soft ferrite recommended in the SAE-J2954 standard are the N96 and PC95 materials of the TDK Company, which are the most widely used high-performance Mn-Zn ferrite materials in the WPT field at the moment.

### 3.3. Amorphous and Nanocrystalline Alloys

Amorphous and nanocrystalline soft magnetic materials mainly include Fe, Ni, Co, Fe-Ni, and Fe-Co-based materials. Amorphous materials are produced by ultra-rapid cooling solidification technology with a cooling rate of approximately 106 °C/s. The thin ribbons of 15–30 μm thick alloys are formed from the liquid state of the metal in a single process. The atoms of amorphous alloys do not crystallize in an orderly arrangement under the effect of rapid cooling. There are no grains and grain boundaries of crystalline alloys, so they show a long-range disorderly arrangement and exhibit isotropic characteristics. This amorphous state has excellent soft magnetic properties, including high permeability, low coercivity, low magnetic loss, and high saturation magnetic induction strength, while the material is strong and wear resistant. Since amorphous alloys are in a thermodynamically nonequilibrium sub-stable state, under appropriate heat treatment process conditions, amorphous alloys crystallize to obtain precipitated crystalline phases with grain sizes below 20 nm, leading to the preparation of nanocrystalline materials or nanocrystalline/amorphous composites [145]. With their excellent magnetic properties, amorphous and nanocrystalline alloys can replace silicon steel, permalloy, and ferrites. They are widely used in many electromagnetic fields such as distribution transformers, sensors, and electromagnetic shielding. With the rise of near-field communication and wireless charging, the application of amorphous and nanocrystalline materials in the field of electromagnetic shielding and WPT is gradually gaining attention.

In the 1970s, researchers developed Fe-Ni-P-B, Fe-Ni-P-B-M, Fe-B, Fe-B-C, Fe-Si-B, Fe-Si-B-M series Fe-based amorphous alloys and Co-based soft magnetic amorphous alloys based on the alloy melt-spin quenching technology [146]. The METGLAS series of Fe-based, Co-based, and FeNi-based amorphous alloy ribbons were produced based on the planar flow casting technology. Since then, soft magnetic amorphous alloys have entered the era of industrialization and commercialization. Among them, Fe-based amorphous alloys have occupied the mainstream of the amorphous alloy industry with their high magnetic properties, low cost, and good amorphous formation ability [147]. The METGLAS 2605SA1(1K101) series FeSiB Fe-based soft magnetic amorphous alloys have been used in large quantities for manufacturing transformer cores and are now gradually used for wireless charging fields.

Nanocrystalline soft magnetic materials have been developed in three systems, Finemet-type, Nanoperm-type Fe-based nanocrystalline alloys, and Hitperm-type FeCo-based nanocrystalline alloys [148]. Their structures and magnetic properties are shown in Table 8. The anisotropy of the average magnetic crystal of nanocrystalline alloys is weak, so the magnetostriction coefficient can be reduced close to zero by adjusting the composition and process [149,150,151]. The permeability and saturation flux density of the alloy can be effectively increased because of the exchange coupling between the amorphous matrix and the nanocrystalline grains [152]. Yoshizawa [153] developed the first five-membered nanocrystalline alloy with a typical composition of Fe_73.5_Si_13.5_B_9_Nb_3_Cu_1_ by adding small amounts of Cu and Nb to FeSiB alloy and registered it as FINEMET alloy. The saturation magnetic induction strength of this material is 1.1–1.5 T, which is slightly lower than that of commonly used amorphous alloys (1.56 T) and silicon steel. It had relatively small losses in the medium and high frequencies and a Curie temperature *T_C_* of 570–600 °C. In the 1990s, Finemet-type nanocrystalline alloys produced by Hitachi Metals in Japan and Vacuum Metallurgical Corporation (VAC) in Germany had matured. Hitachi Metals formed nine FT-1 products, and VAC formed three Vitroperm products [154].

Due to their attractive properties [155], amorphous and nanocrystalline soft magnetic materials have become a hot research topic since their invention [156]. The maximum *B_s_* and *T_c_* characteristics of the material can be significantly enhanced by adjusting the typical element content of amorphous and nanocrystalline materials [157,158,159,160,161,162,163,164,165,166]. Suzuki [167] developed a FeZrB amorphous nanocrystalline duplex alloy system with high Fe content and registered it as the NANOPERM alloy. It is a FeMB-based alloy, where M is Zr, Nb, or Hf in the three-element alloy, and M is further increased by Cu, P, V, and Co in the four or five-element alloys. The *B_s_* of the alloy increases to 1.5–1.7 T, which is higher than that of the FINEMET alloy and almost equal to that of the iron-based amorphous alloy, and its *T_c_* reaches 770 °C. However, it has a high temperature during melting and rapid cooling for ribbon production. Thus, the easy oxidation of Zr, Nb, and Hf elements in the alloy and the requirement for preparation and production under vacuum or gas protection make it difficult for low-cost industrial applications [168].

Willard [169] added Co to create a new variety of Fe-Co-based nanoalloys. The typical composition is a Hitperm-type five-element alloy with Fe_44_Co_44_Zr_2_B_4_C_1_, which precipitates α-FeCo nanocrystalline phase with a grain size of 10–15 nm. However, the cost of this alloy is high due to a large amount of use of the Cobalt. Then, Ogawa [170] invented HB1 iron-based amorphous alloy with *B_s_* reaching about 1.64 T. Makino [171] studied Fe-Si-B-P-Cu nanocrystalline, and its *B_s_* can reach about 1.9 T without the addition of Cobalt precious metal, which dramatically reduces the cost.

The earliest typical application of amorphous and nanocrystalline materials is in motor stators [172,173,174,175,176,177]. Hitachi used laminated amorphous cores in an 11 kW motor prototype with a system efficiency of IE5 efficiency class [178]. In addition, high-efficiency nanocrystalline motor stator cores have been proposed and validated to effectively improve the iron loss of permanent magnet synchronous motors [179,180]. Another typical application of amorphous and nanocrystalline materials is the cores of high-frequency transformers [181,182]. Amorphous iron core distribution transformers have been widely adopted in Asia to reduce grid losses. By the end of 2010, the total capacity of these transformers reached 70 million kVA in China and 35 million kVA in India [183].

Nanocrystalline materials have gained the attention of scholars in the field of WPT in recent years. They have started to apply the matured Fe-based nanocrystalline materials to various wireless charging products, including but not limited to cell phones and electric vehicles [184]. The Samsung Galaxy S6 mobile phone uses ferrite and amorphous soft magnetic sheet as the magnetic core to reduce the phone’s weight and improve its wireless charging efficiency. Starting from the Galaxy S7 series phones, Samsung replaced the previous core material with nanocrystalline. The nanocrystalline core can be compatible with both the NFC function and wireless charging function of cell phones and has achieved good application results, which marked the gradual transition from ferrite to nanocrystalline as the soft magnetic material for mobile phone WPT. In addition to Samsung, more and more brands such as Apple and Huawei have adopted similar nanocrystalline soft magnetic sheet solutions as the magnetic cores for the wireless charging systems of electronic products.

In the field of EV-WPT, Long [185,186,187,188] from the University of Cambridge proposed the use of Hitachi FT-3M type Fe-based nanocrystalline as receiver-side cores in three EV-WPT power levels specified in the SAE-J2954 standard. Among them, in the 11 kW WPT3 system, the system efficiency is improved by 2%, and the coupling coefficient is improved by 13% compared to using TDK’s N87 Mn-Zn ferrite. In the WPT1 and WPT2 systems, the nanocrystalline cores exhibit slightly lower effects than the ferrites but are acceptable. The experimental results show that in the high-power EV-WPT application scenario, the iron-based nanocrystal material exerts its advantages of high saturation magnetic density and low hysteresis loss and can effectively reduce the size and weight of the vehicle end (VA), which has great potential application prospects. Xiong [189] also proposed to use Fe-based nanocrystalline as magnetic cores in the WPT2 and WPT3 EV-WPT systems. The experimental efficiency of the magnetic coupler with the designed core thickness of 2 mm reached 97.4%. The maximum core temperature was about 80.9 °C, and the magnetic flux leakage fully satisfied the ICNIRP 2010 Guidance [190] proposed by the International Commission on Non-Ionizing Radiation Protection. 3M Systems Research Institute developed a 7.7 kW EV wireless charging experimental platform based on Fe-based nanocrystalline cores [191]. Their experimental results showed that using Fe-based nanocrystalline cores can achieve 65% of the weight, 84% of the vertical space volume optimization, and 16% of the electromagnetic leakage of the conventional ferrite platform. The excellent consistency and flexibility of nanocrystals make the system operation more stable and reliable and less prone to damage due to transportation. Jiang [192] compared the electromagnetic characteristics of common mode inductors made from toroidal cores of different soft magnetic materials, which can be applied as compensation inductors in typical LCC-LCC or LCC-S topologies of EV-WPT systems. The hysteresis loss, thermal distribution, and frequency characteristics of Kool Mμ, X Flux, High Flux, MPP, and Fe-based nanocrystalline core common-mode inductors are analyzed in the experiments. It is demonstrated that the electromagnetic temperature characteristics of nanocrystalline and MPP inductors are comparable and that nanocrystalline materials are more advantageous in terms of magnetic heat distribution and electromagnetic performance at operating frequencies above 450 kHz.

**Figure 9:**
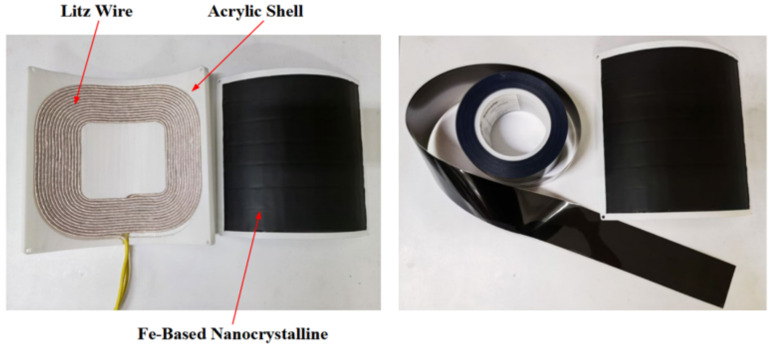
The UUV WPT magnetic coupler and its Fe-based nanocrystalline cores presented in [194].

In addition, the flexible nanocrystalline ribbons allow the cores to generate good deformation forces, making them suitable for applications in certain WPT devices with special shells. Bie [193] from HIT proposed an anti-misalignment UAV wireless charging platform with a solenoidal coil wound on the receiver side of the UAV’s kickstand. The prototype uses nanocrystalline ribbons as magnetic cores and is attached to the kickstand, achieving a conformal design and good transmission efficiency. Wang [194,195] and Cai [196] from HIT proposed the application of Fe-based nanocrystalline ribbons to the UUV WPT system, optimizing the size and weight of the magnetic coupler by about 40% compared to the prototype with ferrite cores. In addition, the system has been designed to be lightweight and conformal. The UUV WPT magnetic coupler and its Fe-based nanocrystalline cores presented in [194] is shown in Figure 9. It is believed that in the early future, nanocrystalline magnetic cores will be increasingly used in aerospace, underwater, and other wireless charging devices with particular fields. Meanwhile, the global annual production of nanocrystalline materials has exceeded 1 million kg and is continuing to increase [156]. Researchers are also continuously working to improve the performance of amorphous and nanocrystalline alloys, especially seeking to increase the maximum saturation flux density [197] to accommodate more high-power, integrated WPT applications.

### 3.4. Comparison of the Typical WPT Materials

The review in the previous two sections shows that ferrite, amorphous and nanocrystalline are the most widely used soft magnetic materials in WPT. Table 9 compares the basic characteristic parameters of several typical soft magnetic materials for wireless energy transfer. The *B_s_* (0.36~0.54 T) of soft magnetic ferrite are low. The saturation flux densities of Fe-based amorphous alloys and nanocrystalline alloys are higher, reaching 1.56 T and 1.35 T. The *T_C_* of Fe-based amorphous alloys and nanocrystalline alloys is higher (400~800 °C), which is less affected by temperature and more adaptable to the working environment.

Nanocrystalline alloys combine the high saturation magnetic induction strength of Fe-based amorphous alloys with the high permeability of Co-based amorphous alloys. They have 2~3 times higher saturation flux density tolerance with lower losses than ferrites below 50 kHz. At 0.2 T/100 kHz, the power loss of the Fe-based nanocrystalline alloy FeCuNbSiB is comparable to that of PC95 ferrite, with the shortcoming that its permeability decreases more rapidly with the increasing frequency. Soft magnetic ferrites have high resistivity and are suitable for medium to high-frequency applications. The Fe-based amorphous alloy FeSiB and the nanocrystalline alloy FeCuNbSiB have a smaller resistivity (120–140 μΩ·cm), but they have excellent overall magnetic properties and still have a place in the medium and high-frequency applications.

The coil will excite a stronger alternating magnetic field as the demand for wireless fast charging increases, meaning greater transmission power. This will lead to an increase in magnetic induction between the primary and secondary sides of the magnetic coupler, which will lead to saturation of the core, a decrease in magnetic permeability, and a weakening of the magnetic coupling effect. In addition, the core losses are proportional to the magnetic induction strength, and magnetic saturation often brings increased losses and heating simultaneously. Ferrite cores are susceptible to magnetic saturation in WPT applications for high power, high integration devices due to the nonlinear magnetic properties of ferrite. One effective solution is to increase magnetic cores, which will undoubtedly increase the weight and size of the system. In addition, ferrite’s brittle and fragile nature makes it unsuitable for electric vehicle onboard receiver applications, where the core structure is easily damaged by driving bumps and flying stones. It will lead to changes in the system self-inductance mutual inductance parameters, which will affect the magnetic coupling resonance state and cause a reduction in the system transmission efficiency.

A comparison of the characteristics of the EV-WPT system using TDK PC-95 Mn-Zn ferrite core and Hitachi Finemet nanocrystalline materials is shown in Table 10. It can be seen that the nanocrystal material is more advantageous in terms of reliability and being lightweight, but its current cost is 3–4 times higher than that of ferrite material. The hysteresis loss of nanocrystal is as good as PC95. Still, its high conductivity will bring some additional eddy current loss, so it is slightly weaker in magnetic coupling performance and magnetic shielding performance. However, it is still within the acceptable range. To conclude, applying nanocrystalline materials in WPT devices is currently gaining momentum. It is believed that as the application of nanocrystalline materials in WPT technology increases and the product preparation process matures, its cost will also gradually decrease to meet the demand for commercial applications in EVs and other devices.

### 3.5. Novel Electromagnetic Metamaterials

Metamaterials are a new class of functional magnetic materials, generally composed of metallic microstructures and resin and ceramic materials with complex artificial designs. It can achieve modulation of electromagnetic fields and waves and change the electromagnetic field distribution and propagation of electromagnetic waves [198]. Metamaterials are generally composed of an array structure of periodic subwavelength structural units. Its design principle is similar to that of crystals with periodic atomic or molecular arrangements in natural materials. The periodic structure of metamaterials interacts with electromagnetic waves to produce a complex electromagnetic response so that the composite material can exhibit extraordinary electromagnetic properties [199].

In essence, the metamaterial is an artificial composite material. With the birth of metamaterials, this novel material design concept has also received widespread attention, marking that human beings can reverse design and prepare new structural materials according to their own will and application requirements through certain technical means and equipment manufacturing means based on the understanding of transformation and utilization of existing materials. This design idea of artificial mordant materials is of great significance in the field of materials technology [200,201,202,203]. The birth and development of electromagnetic metamaterials provide an adequate technical way to artificially regulate electromagnetic fields and waves, break through the physical limits of traditional materials, and enable the application of many extraordinary electromagnetic properties that are impossible to achieve with a single natural material [204,205]. In recent years, researchers have conducted relevant studies and demonstrated the role of metamaterials in magnetic field modulation and tried to apply them in the field of high-frequency WPT (HF-WPT) technology, which has become a new and vital research direction for WPT magnetic materials [206,207,208,209,210,211].

**Figure 10:**
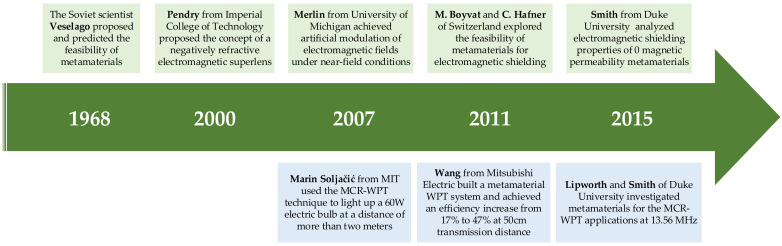
The joint development history of metamaterials and WPT technology.

Both electromagnetic metamaterials and MCR-WPT technologies are newly developed frontier technologies in the 21st century. The Soviet scientist Veselago first proposed the concept of metamaterials. Since 2000, metamaterials have started to develop rapidly under the impetus of Professor Pendry at Imperial College of Technology. In 2007, R. Merlin at the University of Michigan demonstrated the feasibility of achieving artificial modulation of electromagnetic fields under near-field conditions, theoretically deriving and verifying the perfect focusing effect using electromagnetic metamaterials [212]. In the same year, Marin Soljačić [58] from MIT used the MCR-WPT technique to light up a 60 W electric bulb at a distance of more than two meters. Since then, the two frontier technologies, electromagnetic metamaterials and MCR-WPT have developed in parallel and unfolded a combination of technologies. The development history of electromagnetic metamaterial technology is shown in Figure 10.

In 2010, J. Choi and C. Seo of Soongsil University first reported loading an electromagnetic metamaterial with an equivalent permeability of -1 into a near-field MCR-WPT system [213], which theoretically and experimentally demonstrated that the metamaterial has strong electromagnetic field modulation properties. The experimental results show that the WPT system containing metamaterials can achieve a system coupling efficiency of 81.7% at a transmission distance of 1.5 m. Compared with the device without metamaterials, the coupling efficiency was improved by 21%. in 2011, Wang [214] of Mitsubishi Electric built an HF-WPT system containing electromagnetic metamaterials with a resonant frequency of 27 MHz. The transmission efficiency of the wireless transmission system was improved from 17% to 47% at a transmission distance of 50 cm from the conventional structure. In 2015, Lipworth and Smith from Duke University investigated the application of metamaterials in WPT systems with 13.56 MHz resonant frequency [215]. The role of electromagnetic metamaterials in wireless energy transmission systems for magnetic field focusing effects, enhanced mutual inductive coupling, and analysis of electromagnetic loss characteristics of electromagnetic metamaterials are discussed. In addition, scholars from Kyung Hee University [216], Seoul National University [217], KAIST [218], University of Utah [219], and Chulalongkorn University [220] have conducted studies on metamaterial cores for WPT systems at different frequencies, confirming the importance of metamaterials in WPT technology.

In summary, the main service targets of metamaterials are the 6.78 MHz and 13.56 MHz HF-WPT systems under the Alliance for Wireless Power (A4WP) standard. In view of the current research progress, there is still room for optimization and improvement of the transmission efficiency and electromagnetic compatibility of metamaterials HF WPT systems, which is also one of the leading development directions of the technology in the future.

## 4. Critical Issues and Hotspots of Magnetic Materials in WPT

As mentioned in the introduction part, magnetic materials usually play two significant roles in WPT systems to enhance coupling performance and achieve electromagnetic shielding. However, few existing designs and studies for magnetically coupled mechanisms have reported combining magnetic materials with coils for simultaneous design. The general design flow of the magnetic coupler is to add magnetic material with about the same size as the coil to the system under the premise of determining the structure and size of the coil and studying the effect of electromagnetic parameters and the shape of the magnetic material on the efficiency of the system. However, it is obvious that this design flow is contrary to the magnetic device design idea. Therefore, this section will discuss and analyze several critical issues and research hotspots concerning the magnetic material and core structure of the magnetic coupler of the WPT system up to now and try to give some design ideas that may be constructive.

### 4.1. Aggregated Magnetic Coupler Design

**Figure 11:**
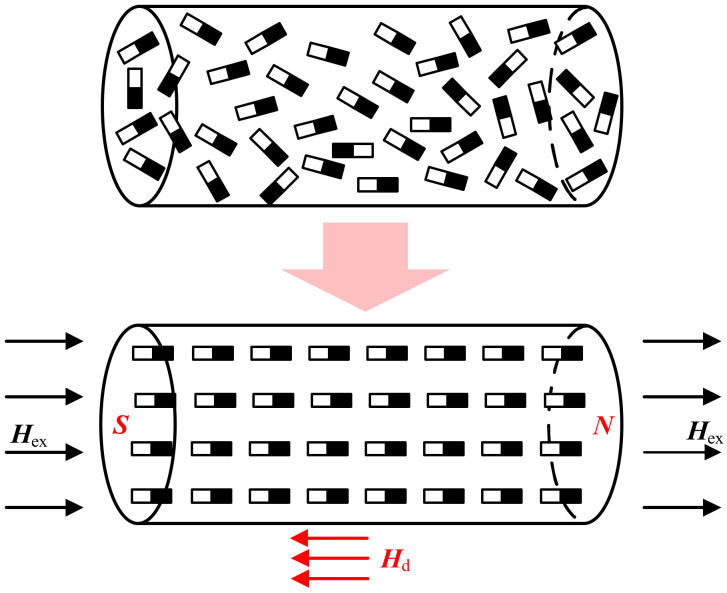
Schematic diagram of the magnetization principle of the magnetic core.

The magnetic material will be magnetized under the action of the external magnetic field, and the generated demagnetization magnetic field will react to the external magnetic field, which will affect the intensity and distribution of the magnetic field. The relationship between the coupling performance and magnetic core structural parameters and magnetization can be revealed through the analysis of the magnetic core magnetization microscopic mechanism. The microscopic mechanism of magnetization and demagnetization of the magnetic core is shown in Figure 11, where *H_ex_* is the external magnetic field and *H_d_* is the demagnetization magnetic field. When there is no *H_ex_*, the orientation of the magnetic dipoles inside the medium is disordered, and their magnetic dipole moments cancel each other out. Macroscopically, they do not show magnetism to the outside, and the magnetic core is in unmagnetized. On the other hand, when there is an external magnetic field, the magnetic dipoles will be aligned along the direction of the external magnetic field to form a magnetized and demagnetized magnetic field. The effective magnetic field strength inside the magnetic core can be expressed as *H_eff_* = *H_ex_* − *H_d_*. When the magnetic core is uniformly magnetized, the demagnetization magnetic field strength *H_d_* = *F* × *M*, where *F* is the demagnetization factor and *M* is the magnetization intensity. Existing research shows that the magnetization *M* is only related to the material of the magnetic core, and the demagnetization factor *F* depends on the geometric structure and orderly arrangement of the magnetic core. Therefore, the structure and parameters of the magnetic core can be designed by establishing the relationship between the microscopic demagnetization factor and the macroscopic magnetic core size parameters.

**Figure 12:**
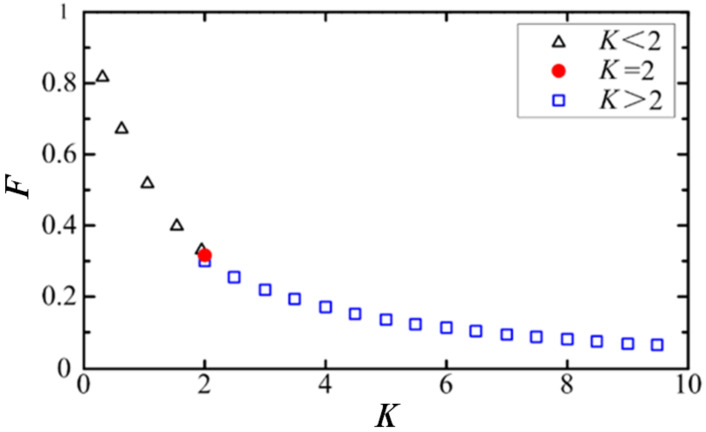
Schematic diagram of the magnetization principle of the magnetic core.

For a general strip core structure, the relationship between the demagnetization factor and core geometry parameters is shown in Figure 12. Where *K* is the ratio of core length to the cross-sectional radius. It can be seen that F decreases as the core length increases. This is because as the core length increases, the distance between the north and south poles of the demagnetizing field increases, which increases the path of the demagnetizing magnetic lines and weakens the demagnetizing field.

**Figure 13:**
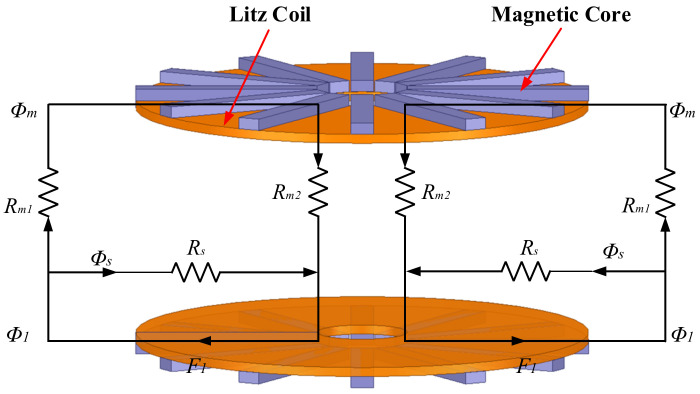
Planar circular magnetic coupler reluctance model.

The reluctance model combined with the magnetic coupler can explain the above analysis more efficiently. Figure 13 is the schematic diagram of the reluctance model of the planar circular coil magnetic coupler. *R_s_* is the self-coupling reluctance, *R_m1_* and *R_m2_* are the mutual coupling reluctances, *Φ_s_* is the self-coupling flux, *Φ_m_* is the mutual coupling flux, and *F_1_* is the magnetic potential generated by the transmitter coil. The magnetic flux of the coil of the planar magnetic coupler is symmetrically distributed. Then, according to the equivalent magnetic circuit model, the expression of the basic parameters of the magnetic coupler is as follows:

(1) $$\left\{\begin{array}{l} M=\frac{N\phi_{m}}{i}=\frac{2N^{2}}{R_{m1}+R_{m2}}\\ L_{l}=\frac{N\phi_{l}}{i}=\frac{2N^{2}}{R_{s}}\\ L_{2}=L_{l}+M\\ k=\frac{\phi_{m}}{\phi_{l}}=\frac{R_{s}//\left(R_{m1}+R_{m2}\right)}{R_{m1}+R_{m2}}=\frac{1}{1+\frac{R_{m1}+R_{m2}}{R_{s}}} \end{array}\right.$$

**Figure 14:**
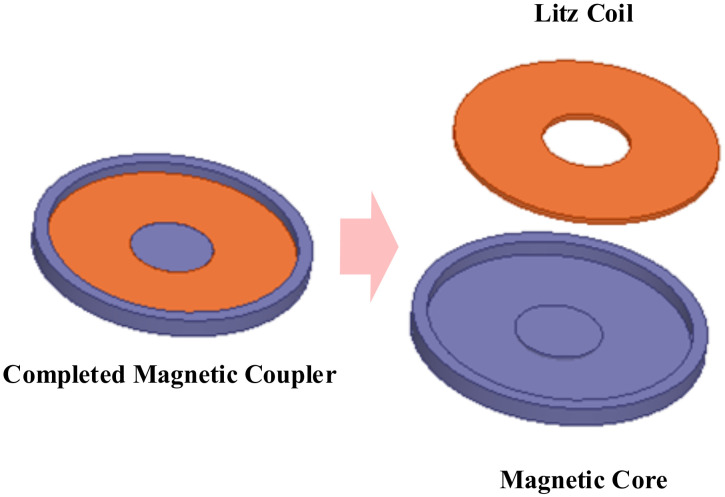
Magnetic circuit optimization of the magnetic coupler.

**Figure 15:**
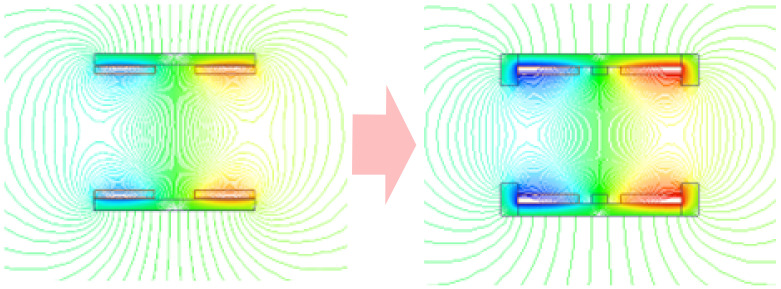
Distribution of magnetic field lines before and after aggregated optimization.

It can be seen that reducing *R_m1_* and *R_m2_* is the key to improving the coupling ability of the WPT system. In addition, the most direct and effective way to minimize reluctance is to add magnetic cores. For a planar magnetic core, the magnetic field lines generated by its magnetization are not closed inside the magnetic core. So, the demagnetization magnetic field will also be generated when the magnetic core is magnetized, and the demagnetization magnetic field will affect the magnetic circuit characteristics of the magnetic coupler. In order to reduce the influence of the demagnetization magnetic field, the central cylinder and the outer protrusion of the magnetic core improved design extend the path of the demagnetization magnetic field lines and increases the coupling degree of the magnetic coupler. The optimized structure of the magnetic coupler from Figure 13 is shown in Figure 14. At the same time, the protrusion on the outer side of the magnetic core constrains the leakage magnetic field outside the magnetic coupler, which plays a role in gathering the magnetic field and reducing the magnetic leakage. Furthermore, when this coupler structure is applied in the scenario of planar array coils, the cross-coupling between adjacent coils can also be effectively reduced. The distributions of the magnetic flux lines are shown in Figure 15. It can be seen that the design of the cylinder in the middle of the magnetic core and the outer protrusions effectively extend the demagnetization magnetic field lines and enhance the internal magnetic field strength of the coupler.

The above analysis provides an idea for the integrated design of the magnetic aggregation magnetic coupler. The relationship between the demagnetizing magnetic field, coupling coefficient, and core structure parameters is established through the finite element and analytical calculations. Then, the optimization of the magnetic coupler is achieved by using the characteristics of the influence of the magnetic material structure on the demagnetizing magnetic field and the degree of coupling. However, on the contrary, the increase in the core geometry will increase the size and weight of the magnetic coupler. So, the design process should also seek the balance between the size, weight, power density, transmission performance, and cost of the magnetic coupler.

### 4.2. Modeling of the MC under Finite Size Core Conditions

Until today, the accurate analytical modeling of magnetic couplers is still a key and hot issue in WPT technology. Precise modeling of WPT magnetic couplers allows the solution of system coupling performance, coil resistance, self-inductance, and coupling coefficients without the use of finite element software at the early design stage. This is very helpful in quickly determining whether the design parameters of the magnetic coupler meet the expectations in the early design stage. The modeling and analytical calculations are easier for hollow circular coil couplers without magnetic cores. Ref. [221,222] accurately calculated the mutual inductance of a circular planar coil in the air by dividing the circular planar coil into n-equivalent polygons, which has the advantage of being able to predict the mutual inductance of an n-sided planar coil, but this method is more computationally intensive when n is larger. Ref. [223] calculates the mutual inductance of two planar coils horizontally offset in the air by magnetic vector potential. Ref. [224] derived an approximate expression for the mutual inductance of a horizontally offset cylindrical thin-walled coil in air and between rings using a known elliptic integral. Ref. [225,226] proposed an improved algorithm for mutual inductance between coils at arbitrary spatial locations based on Neumann’s formula. An analytical model based on the Biot–Savart law approximates the mutual inductance of circular and square planar coils at arbitrary positions in the air [227].

Since magnetic couplers usually contain finite size magnetic cores and aluminum shield plates, the above analytical calculation method will no longer apply. There is no doubt that magnetic coupler modeling under finite size core conditions is a hot research topic in the WPT technology. Ref. [228] used the current mirror method to calculate the mutual inductance between circular coils on a single-sided single-layer infinite size magnetic shielding material. Luo [229] modeled the magnetic coupler of the WPT system in the cylindrical coordinate system using Maxwell’s equations. Combined with the Fourier–Bessel transform, the solution of Poisson’s equations was simplified and derived an analytical method to calculate the mutual inductance of square and circular coils. However, the model is developed under the conditions of an ideal infinite size magnetic core. Ref. [230] proposed a new analytical method for mutual inductance calculation based on the electric field strength and coordinate transformation method. However, this method is also implemented under the assumption of infinite magnetic materials, so the accuracy of its calculation results may not be considered high. In the latest research progress, Li proposed a novel analytical calculation method for the WPT magnetic coupler under the conditions of an infinite size core and single-sided single-layer finite-size core in [231,232], respectively, which provides a feasible analytical modeling idea.

**Figure 16:**
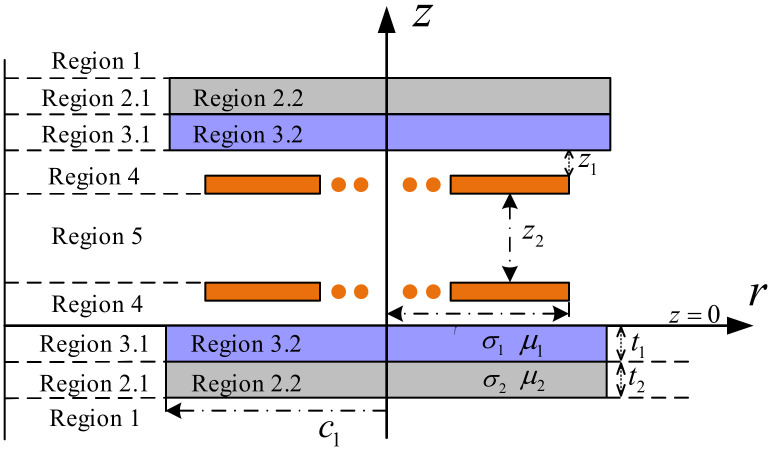
Distribution of magnetic field lines before and after optimization.

The two-dimensional geometric model of a planar MC is shown in Figure 16. Assume that the regions are linear, homogeneous, and isotropic, then the electromagnetic field can be expressed based on Maxwell’s equations. The magnetic vector potential ***A*** can be expressed as ***A****_α_* (*α* = 1~5) according to different regions, where ***A****_2_* and ***A****_3_* can be divided into ***A****_2.1_*, ***A****_2.2_* and ***A****_3.1_*, ***A****_3.2_*. The Poisson equation of the magnetic vector potential ***A*** in each region can be expressed as:

(2) $$\nabla^{2}A_{\alpha }+\left(k^{2}\left(\alpha \right)-\frac{1}{r^{2}}\right)A_{\alpha }=0$$

where $k^{2}\left(\alpha \right)=-j\omega \mu_{\alpha }\sigma_{\alpha }$.$\sigma_{\alpha }$ and $\mu_{\alpha }$ represent the electrical conductivity and magnetic permeability of each dielectric layer, respectively.

Since the conductivity of the air layer is zero, there are $k^{2}\left(1,2.1,3.1,4,5\right)=0$, $k^{2}\left(2.2\right)=-j\omega \mu_{1}\sigma_{1}$, and $k^{2}\left(3.2\right)=-j\omega \mu_{2}\sigma_{2}$. It is worth noting that the Poisson equation of the magnetic vector potential A in each region is obtained by bringing the Coulomb gauge condition $\left(\nabla \cdot A=0\right)$ into Maxwell’s basic equation. Whether the number of layers and the size of the dielectric layer is limited does not affect the Poisson equation in each region. Applying the first-order Fourier–Bessel integral transform to solve the Poisson equation, the general solution of the magnetic vector potential A in each region can be obtained as:

(3) $$A\left(r,z\right)=\sum_{i=1}^{\infty }\left[A_{i}J_{1}\left(k_{i}r\right)+B_{i}Y_{1}\left(k_{i}r\right)\right]\cdot \left[C_{i}e^{\lambda_{i}z}+D_{i}e^{-\lambda_{i}z}\right]$$

where *k_i_* and *λ_i_* are the eigenvalues, and $\lambda_{i}=\sqrt{k_{i}^{2}+j\omega \mu_{0}\mu_{r}\sigma }$. *J_1_*, *Y_1_* represent the Bessel function of the first kind and the Bessel function of the second kind, respectively. The unknown coefficients *A_i_*, *B_i_*, *C_i_*, *D_i_* in the general solution can be obtained by using the boundary conditions of the interface in the solution domain.

Furthermore, according to the Nierman formula, the expression of the flux linkage can be obtained by the line integral of the magnetic vector potential, and then the self-inductance *L* and mutual inductance *M* of the coupling mechanism can be obtained, and the expressions are as follows:

(4) $$\left\{\begin{array}{l} L=2\pi \sum_{p=1}^{n_{t}}\sum_{p^{\prime }\neq p}^{n_{t}}r_{p}A_{p^{\prime }}\left(r_{p},0\right)+2\pi \sum_{p=1}^{{n^{\prime }}_{t}}r_{p}A_{p}\left(r_{p}-r_{c},0\right)\\ M=2\pi \sum_{p=1}^{n_{t}}\sum_{q=1}^{n_{t}{}^{\prime }}r_{q}A_{p^{\prime }}\left(r_{q},z_{2}\right) \end{array}\right.$$

where *r_p_* and *r_q_* represent the radius of the *p*-th coil and the *q*-th coil respectively. z2 is the distance between the transmitting coil and the receiving coil. *n_t_* and ${n^{\prime }}_{t}$ are the number of turns of the transmitter and receiver coil, *r_c_* is the diameter of the Litz wire. Under high-frequency operating conditions, the AC internal resistance of the Litz wire is mainly composed of conduction resistance and proximity effect resistance, and its expressions are respectively:

(5) $$\left\{\begin{array}{l} R_{cond}=\frac{\xi }{n_{s}2\pi r_{s}^{2}\sigma }\Phi_{cond}\left(\xi r_{s}\right)\sum_{p=1}^{n_{t}}2\pi a_{p}\\ R_{prox}=\frac{-4\pi n_{s}\xi r_{s}}{\sigma }\Phi_{prox}\left(\xi r_{s}\right)\sum_{p=1}^{n_{t}}2\pi a_{p}\langle H^{2}\left(i\right)\rangle \end{array}\right.$$

where *n_s_* and *r_s_* are the number of strands and the radius of a single strand of the Litz wire, respectively. $\langle H^{2}\left(i\right)\rangle$ is the square of the average field strength on the cross section of the *i*-th coil. σ is the electrical conductivity of the Litz wire. $\xi =\sqrt{2}/\delta =\sqrt{2\pi \mu_{0}\sigma f}$, *μ_0_* is the magnetic permeability of air and *δ* is the skin depth of the coil, *f* denotes the frequency of the excitation current in the transmit coil. $\Phi_{cond}\left(\xi r_{s}\right)$ and $\Phi_{prox}\left(\xi r_{s}\right)$ are the Kelvin function.

Obviously, the above analysis is more general than the previous studies and can be extended to the particular conditions of misalignment between coils. Analytical modeling of electromagnetic problems using Maxwell’s equations is a classical tool that has been applied extensively in the study of magnetic field problems under stratified conditions in electromagnetic media. In the last decade, existing research has also been gradually improving the original classical theory. Ref. [229] proposed a double Fourier transform theory, which greatly simplifies the problem of solving Poisson’s equation in the Cartesian coordinate system. In the field of eddy current non-destructive testing (NDT), Ref. [233] proposed a truncated region eigenfunction expansion (TREE) method to calculate the potential magnetic vector of a circular coil with a circular magnetic medium. It marks the beginning of the researchers’ study of the magnetic field problem in the presence of finite size magnetic medium stratification. In [234], a unique source function is invoked on the basis of the TREE method to simplify and speed up the calculation process in the [233], and an approximate formula for the mutual inductance of a circular coil with a circular magnetic core is obtained. Dong [235] used the TREE method to analytically calculate the mutual inductance with a reverse series coil coupler and optimize the system parameters based on their analytical calculation results. The radius and number of turns of the special coils are determined quickly and precisely with guaranteed magnetic field uniformity.

It can be seen that the classical Maxwell modeling method still has a large number of applications in the analysis, optimization, and design of magnetic field problems today. For a long time, isotropic soft magnetic materials represented by Mn-Zn ferrite are still the preferred choice for WPT magnetic coupler cores. The above-mentioned analysis method can intuitively determine the degree of influence of the magnetic coupler parameters on the system characteristics. However, with the continuous development of WPT electromagnetic shielding technology, more and more embedded and composite magnetic materials are used as magnetic cores, which is undoubtedly a challenge for the present method. Fortunately, with the use of the homogeneous medium analysis method [236], the inhomogeneous core structure could be simplified into an equivalent model that can be analyzed analytically. Moreover, the analysis and simulation of the complex WPT structure can be achieved with the help of powerful FEM software.

### 4.3. Electromagnetic Shielding of WPT

Electromagnetic environment safety is a problem that must be solved in promoting the application of wireless energy transmission technology. Electromagnetic shielding technology can improve the system transmission performance while ensuring the safety of WPT system leakage, which is the current research hotspot of WPT technology. Many authorities have issued electromagnetic safety standards for WPT, setting the contact limits for electric field strength and magnetic field strength according to frequency bands and power levels. Among them, the most famous ones are ICNIRP 1998 and ICNIRP 2010 guidelines developed by the International Commission on Non-Ionizing Radiation Protection (ICNIRP) [190,237] and IEEE Std C95.1-2005 standard set by the Institute of Electrical and Electronics Engineers (IEEE) [238]. Table 11 summarizes the limits of each standard regarding electric field strength, magnetic field strength, and magnetic flux density in the dominant operating range (10 kHz–10 MHz) of WPT systems.

The electromagnetic shielding design of WPT systems can be evaluated in terms of shielding effectiveness [239], which can be defined as:

(6) $$S_{E}=20\mathrm{lg}\left|\frac{B_{z,\;noshielding\;}}{B_{z,\;withshielding\;}}\right|=20\mathrm{lg}\left|\frac{H_{z,\;noshielding\;}}{H_{z,\;withshielding\;}}\right|$$

where **B**_z_ is the magnetic flux density and **H**_z_ is the magnetic field strength of the observed point. Schelkunoff [240] proposed a method to predict the electromagnetic shielding effectiveness of magnetic materials. It was considered that the shielding energy efficiency could be expressed as *S_E_* = *R* + *A* + *R_r_*, where *R* is the reflection loss of the shield, *A* is the absorption loss of the shield, and *R_r_* is the reflection loss inside the shield.

**Figure 17:**
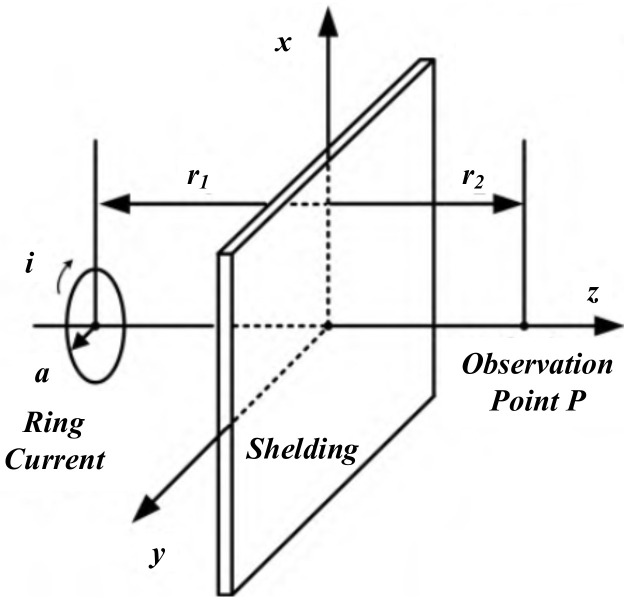
Electromagnetic shielding effectiveness analysis diagram of Moser’s theory.

In 1967, Moser [239] presented an analytical expression for the shielding effectiveness by deriving the exact solution of the vector dynamic potential equation, as shown in Equations (7) and (8). Where *a* is the coil radius, *μ_r_* is the relative permeability of the shield, *σ* is the electrical conductivity of the shield, *c* is the propagation velocity of light in air, *J_1_* is a first-order Bessel function, and *C* is a function concerning *k* related to the thickness and relative permeability of the shield. In addition, using the shielding transmission theory, expressions for the magnetic induction strength and electric field strength at different positions of the metal plate were derived, as shown in Figure 17 and Equations (9) and (10). The evaluation method of shielding effectiveness proposed by Moser is used to this day.

(7) $$S_{E}=\mathrm{lg}\frac{1}{4\mu_{r}}\left|\frac{\int_{0}^{\infty }\frac{k^{2}}{\tau_{0}}J_{1}(ka)e^{-\tau_{0}z}dk}{\int_{0}^{\infty }\frac{Ck^{2}\tau }{\tau_{0}}J_{1}(ka)e^{-\tau_{0}z-t\left(\tau -\tau_{0}\right)}dk}\right|$$

(8) $$\left\{\begin{array}{c} \tau_{0}=\sqrt{k^{2}+\gamma_{0}^{2}}\\ \tau =\sqrt{k^{2}+\gamma^{2}}\\ \gamma_{0}=j\frac{2\pi f}{c}\\ \gamma ≅\sqrt{j\omega \mu_{0}\mu_{r}\sigma } \end{array}\right.$$

(9) $$B=\frac{\mu aI}{2}e_{\rho }\int_{0}^{\infty }\lambda J(\lambda a)J(\lambda \rho )\left(e^{-\tau |z|}-Ce^{\tau z}\right)d\lambda +\frac{\mu aI}{2}e_{z}\int_{0}^{\infty }\frac{\lambda^{2}}{\tau }J(\lambda a)J(\lambda \rho )\left(e^{-\tau |z|}+Ce^{\tau z}\right)d\lambda$$

(10) $$E=-\frac{j\omega \mu aI}{2}e_{\varphi }\int_{0}^{\infty }\frac{\lambda }{\tau }J(\lambda a)J(\lambda \rho )\left(e^{-\tau z}+\right.\left.Ce^{\tau z}\right)d\lambda$$

The shielding methods of WPT can be divided into two categories, active shielding, and passive shielding. The principle of active shielding is to restrain the leakage field with the help of additional shielding coils. Active shielding methods can be further classified into reactive resonant shielding and active coil shielding according to the type of coils added. The current induced by the leakage field in the shielded coil generates a reverse counteracting magnetic field, which cancels the leakage field and thus achieves the shielding purpose [241,242]. This is the principle of reactive resonant shielding, which does not require any additional power supply. By controlling the capacitance connected to the shielding coil, the impedance is optimally matched to reduce the leakage field strength effectively. On the contrary, the active shielding method is to add power to the shielding coil, which is able to achieve electromagnetic shielding for high power level WPT systems with more robust shielding effectiveness [243,244].

The principle of passive shielding is to achieve magnetic field shielding and electric field shielding of the magnetic coupler coil of the WPT system using magnetic materials (Mn-Zn ferrite, nanocrystals, metamaterials) or conductive metal materials (aluminum plates), which is the most common and easiest shielding method to implement. The principle of magnetic field shielding is to use magnetic materials to direct the magnetic flux along the path close to the coupling coil, which improves the coil’s mutual and self-inductance, ultimately achieving a reduction in the leakage magnetic field strength of the WPT system [245]. The principle of electric field shielding is to place a metal plate added to the magnetic coupler. When the high-frequency magnetic field passes through the conductive metal plate, the induced electromotive force generated on the plate is short-circuited by the plate thereby causing an eddy current, which produces a reverse magnetic field and counteracts the leakage magnetic field [246].

Often magnetic field shielding and electric field shielding are used in combination. For example, the SAE-J2954 standard recommends an electromagnetic shielding structure with a combination of ferrite cores and aluminum plates. In general, the aluminum plate is usually close to the secondary side of the receiving end of the housing, the same shape as the receiving end of the housing and is slightly larger than the size of the coil. There is a gap between the aluminum plate and the core to reduce the eddy current effect. In the MC, the core is placed as close as possible to the coil (under the premise of ensuring insulation) to improve the coupling inductance of the coil while also enhancing the shielding effectiveness.

**Figure 18:**
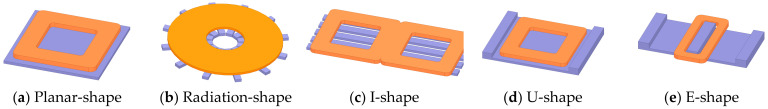
Typical magnetic core shapes of MCR-WPT couplers.

In addition to the magnetic core material type, the core shape is also one of the important factors affecting core performance. Typical core shapes are planar-shape [247,248,249,250,251], radiation-shape [52,252], I-shape [187,253], U-shape [254], and E-shape [255], as shown in Figure 18. Planar cores are the most common structures, while radial and strip cores are lighter structures of planar types. In addition, U-type and E-type as the special core structures are often used to achieve the conformal design. In addition, in some applications with high EMC requirements, active shielding, reactive resonance shielding, and passive shielding can be used in combination to enhance the electromagnetic shielding capability of the system to the maximum extent. The applicability and potential shortcomings of electromagnetic shielding methods applied in MCR-WPT are summarized in Table 12.

### 4.4. Magnetic Core Structure Optimization

The commonly used high permeability nanocrystals tend to exhibit additional core eddy current losses when applied to high-power WPT systems, which are namely detrimental to the efficient power transmission of the system. In addition, little research has been reported on the magnetic structure of WPT systems. Most WPT system cores are composed of a single magnetic material. In fact, the optimization of the magnetic core structure can also achieve the suppression of eddy current loss and the improvement of electromagnetic performance. Therefore, the design and optimization of the electromagnetic structure of the magnetically coupled mechanism is a potential research focus for WPT technology. The optimized design ideas of the magnetic structure of WPT will be given from the following three points.

#### 4.4.1. Refinement Crushing Process and Staggered Arrangement

**Figure 19:**
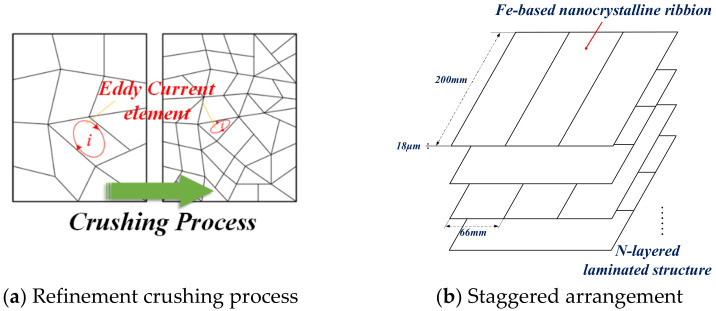
(**a**,**b**) Two processes to reduce additional eddy current loss in nanocrystalline cores.

Refinement Crushing Process and Staggered Arrangement are the unique improvement processes proposed for the performance enhancement of nanocrystalline materials for application in WPT systems [189]. The principle is to improve the resistivity of nanocrystalline strips and suppress their eddy current losses by filling the nanocrystalline strip planes with insulating particles, as shown in Figure 19a. The gap between nanocrystal particles becomes larger after refinement and crushing, and the eddy current element area of nanocrystalline becomes smaller. The finer the grain size, the more pronounced the eddy current blocking effect and the higher the resistivity. Refinement of nanocrystalline ribbons can be achieved by the heat treatment and mechanical crushing.

Unfortunately, the crushing process increases the nanocrystal resistivity and also leads to a decrease in nanocrystal magnetic permeability. However, for the general planar magnetic coupler of the WPT system, when the permeability of the core material is higher than 1000, the increase of permeability will become less evident for the improvement of system self-inductance mutual inductance and coupling coefficient. Therefore, a compromise between two crucial parameters, resistivity, and permeability, is required in the design.

In addition, the loss of nanocrystalline alloy is related to the thinness of each layer. The thinner the Fe-based nanocrystalline alloy is, the lower the magnetic loss in a high-frequency environment. In addition, the splicing gap plays a different role in blocking the eddy currents in the plane of the nanocrystalline core from the macroscopic point of view. Thus, the cores can be further fabricated using the staggered arrangement process, as shown in Figure 19b, which can further reduce the eddy current loss to a certain extent.

#### 4.4.2. Laminated Arrangement Direction

Usually, in order to avoid the magnetic saturation of WPT cores, iron-based nanocrystal layers are required to increase the capacity of the cores. However, the presence of adhesives between the layers of the iron-based nanocrystal laminate structure will lead to changes in the electromagnetic properties of its cores. Since Fe-based nanocrystalline materials have electromagnetic properties of high magnetic permeability and high electrical conductivity, adding adhesives will cause the core to be cut along the normal magnetic path and affect the continuity of the magnetic flux distribution. In other words, the cores have different electromagnetic properties in the tangential and normal directions when a planar laminate stack structure is used.

For the interior of nanocrystalline cores, the electromagnetic properties are almost isotropic. However, for the whole nanocrystalline cores with laminated structures, the electromagnetic properties of the cores show anisotropic behavior externally. The homogeneous medium analysis method can equate the laminated core structure to an anisotropic core monolith for more convenient analysis of the electromagnetic properties of laminated cores.

**Figure 20:**
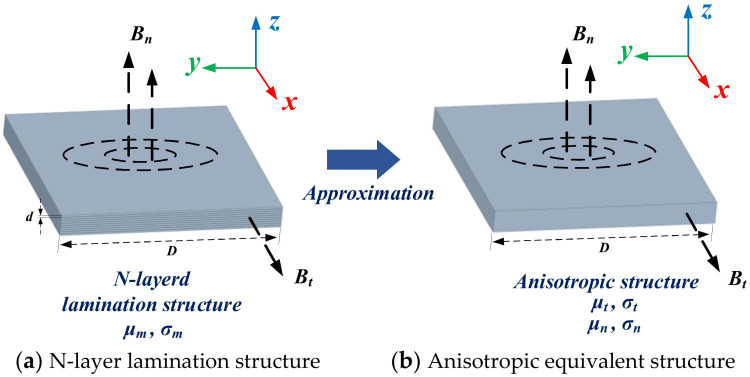
(**a**,**b**) Homogeneous medium approximation method for the magnetism of laminated materials.

Figure 20a is the multi-layered nanocrystalline shielding core, the permeability and conductivity of the Fe-based nanocrystalline material are *μ_m_* and *σ_m_*, while the permeability of the adhesive layer between the ribbons is assumed to *μ_a_*, and the permeability and conductivity of free space are zero. Figure 20b is the equivalent core structure using the homogeneous medium analysis method. The core is a monolithic solid structure, but this model uses separate permeability and conductivity parameters *μ_t_*, *σ_t_* and *μ_n_*, *σ_n_* in the normal and tangential directions to characterize the anisotropy of the laminated structure. Assuming that the fill factor *F* of the laminated core is defined as the total thickness of the Fe-based nanocrystalline ribbon per unit core thickness, the equivalent core structure model parameters can be calculated according to Equations (11)–(14).

(11) $$\mu_{t}=F\mu_{m}+(1-F)\mu_{0}$$

(12) $$\mu_{n}=\frac{\mu_{m}\mu_{0}}{F\mu_{0}+(1-F)\mu_{m}}$$

(13) $$\sigma_{t}=F\sigma_{m}$$

(14) $$\sigma_{n}={\left(\frac{d}{D}\right)}^{2}\frac{1}{F}\sigma_{m}$$

where *d* and *D* are the thickness and width of the laminated Fe-based nanocrystalline ribbons, respectively. In addition, the effective skin depth of the laminated core can be calculated by:

(15) $$\delta_{\mathrm{nano}\;,eq}=\sqrt{\frac{1}{\pi f\mu_{0}\mu_{n}\sigma_{t}}}$$

**Figure 21:**
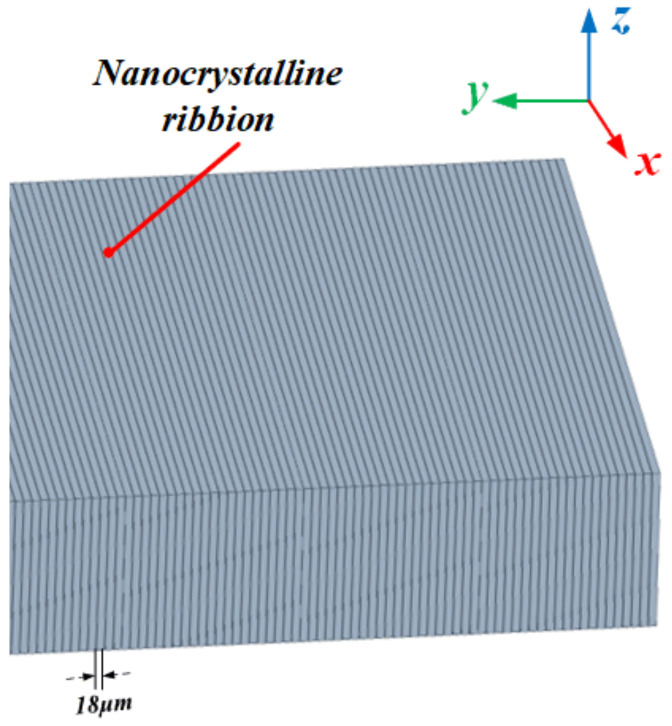
Vertical arrangement of the lamination Fe-based nanocrystalline core.

Therefore, compared with ferrite cores, the magnetic flux distribution within the cross-sectional area of nanocrystalline cores is not uniform. Since the permeability in the parallel and perpendicular directions is very different with respect to the ribbon, the parallel direction (*x*-axis) is the preferred magnetic path for the magnetic flux. The vertical direction (*y*-axis) represents a high reluctance path due to the ribbons between the adhesives [187]. Therefore, when using nanocrystalline materials as the magnetic coupler cores, the vertical arrangement, as shown in Figure 21, can be considered to adapt to the magnetic circuit direction of the circular or DD coils.

#### 4.4.3. Embedded and Composite Magnetic Materials

An attractive novel core structure in the latest research progress is the consideration of ferrite and nanocrystalline for embedding together as the magnetic core. In the saturation-prone region of the core (coil coverage area and main flux path), high-performance nanocrystalline is used to enhance the system coupling capability and anti-saturation capability and to increase the maximum power tolerance of the system. Other areas are covered with ferrite to reduce the additional eddy current loss.

**Figure 22:**
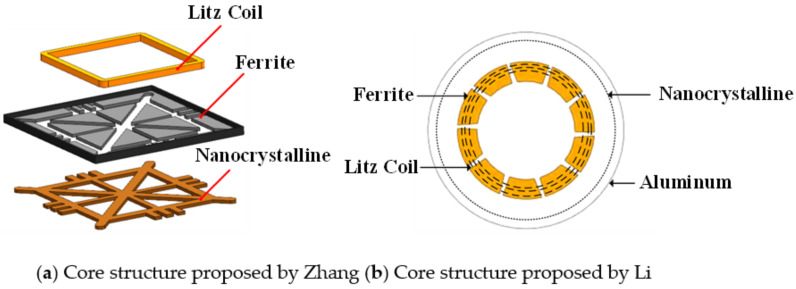
(**a**,**b**) Two types of embedded magnetic structures with nanocrystalline and ferrite.

To solve the problem that a single core cannot meet the electromagnetic safety requirements of the system and the additional eddy current loss in nanocrystalline, Zhang [273] proposed a combined nanocrystalline alloy and ferrite material shielding structure, as shown in Figure 22a. This scheme not only improves the electromagnetic shielding effect but also reduces the adverse effect of eddy current loss in the core material on the system power transmission efficiency. Li [274,275] proposed an electromagnetic shielding structure by adding nanocrystalline material between the sector ferrite and the aluminum plate, as shown in Figure 22b. This structure improves the system coupling coefficient by 7%, while the leakage magnetic field strength in the horizontal direction is reduced by 58% compared to that without shielding.

The embedded magnetic structure can theoretically improve the electromagnetic shielding capability and magnetic coupling performance of the system. However, it is easy to find that this magnetic structure is very complicated to implement. The proposed embedded magnetic structure represents the desire of WPT technicians to improve the existing magnetic materials. There is no doubt that proposing a novel composite magnetic material developed explicitly for WPT technology is the optimal way to solve the above-mentioned set of problems.

### 4.5. Discontinuities of Magnetic Core Channel

Currently, the core materials used in wireless power transmission systems are ferrite or nanocrystal products of certain types from magnetic core companies. Since these cores are not specifically manufactured for WPT systems, they are prone to discontinuous core distributions in the magnetic pathway or the direction of the magnetic field lines during magnetic coupler design. In general, the conductivity and magnetic permeability of magnetic materials are much higher than that of air media. Therefore, the discontinuous distribution of magnetic cores (usually air gap) will potentially lead to the failure of the magnetic coupler model in Section 4.1, resulting in a loss of system design accuracy. In addition, this is more obvious in the design of magnetic couplers with nanocrystalline cores with high electromagnetic performance. The increase of reluctance caused by the discontinuous core distribution and the optimization of the magnetic material on the magnetic coupling performance contradict each other, thus weakening the improvement of the coupling performance of the system by magnetic materials.

**Figure 23:**
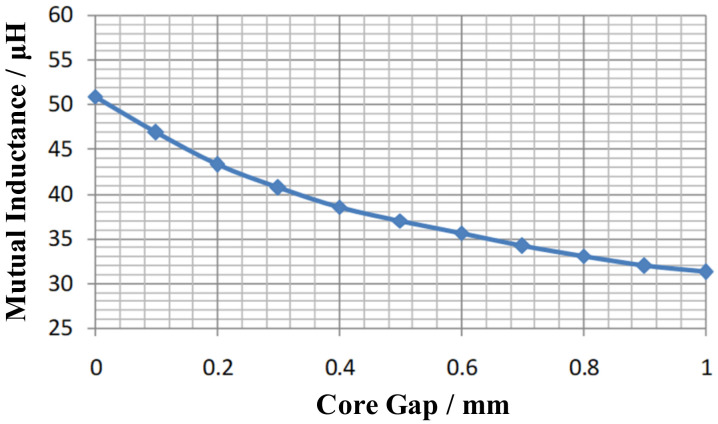
Variation trend of mutual inductance with the core gap.

Figure 23 is a schematic diagram of the finite element simulation relationship between the mutual inductance and the core gap for a similar planar magnetic coupler in Figure 7. It can be seen that the mutual inductance of the magnetic coupler decreases as the core gap increases. In addition, the core gap on the magnetic circuit channel may change during operation due to influencing factors such as temperature, external environmental stress, and magnetostriction, resulting in fluctuations in the mutual inductance of the system. This may have a non-negligible effect on the resonance state, output characteristics, and operational reliability of the WPT system.

**Figure 24:**
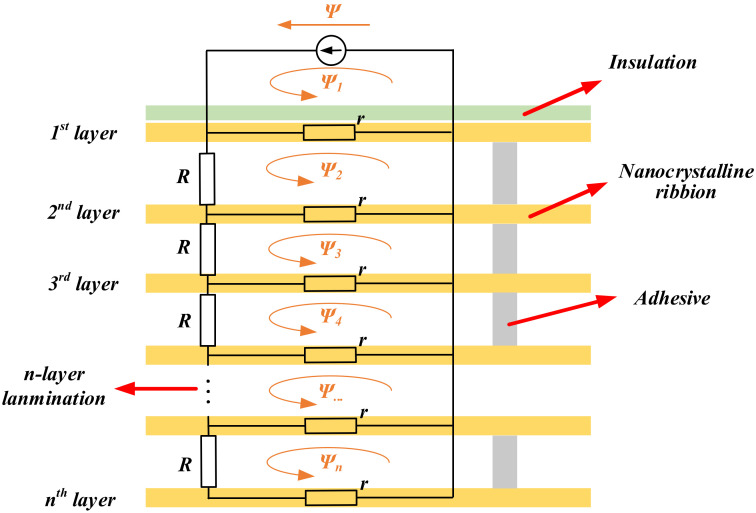
Flux distribution model for laminated core structure.

In addition to affecting the system parameters, discontinuities in the cores can introduce regional saturation problems in the magnetic couplers. This is more pronounced in thinner nanocrystalline ribbon laminated cores [187]. The magnetic flux and reluctance distributions entering the *n*-layer laminated magnetic core structure are shown in Figure 24. *r* and *R* represent the reluctance of the Fe-based nanocrystalline ribbons and the interlayer adhesive, respectively. *ψ* represents the magnetic flux generated by the transmitter coil and entering the Fe-based nanocrystalline core material, and *ψ_1_* >> *ψ_2_* >> *ψ_3_* >> *ψ_4_*. Since *R* >> *r*, the first layer of nanocrystalline ribbon closest to the coil will absorb most of *ψ*, and subsequent ribbons will carry less and less flux.

The distributed saturation effect of magnetic cores needs to be viewed in two ways. On the one hand, a core structure of sufficient capacity and as continuous as possible should be designed in the saturation-prone region of the magnetic coupler to meet the maximum saturation flux density limit and avoid the occurrence of magnetic saturation. On the other hand, in the region where saturation is not desirable, the amount of magnetic material can be reduced to achieve a lightweight design.

## 5. Trends and Opportunities of WPT Magnetic Materials

### 5.1. Proactive Response to The Technology Demands

To be fair, the development of wireless power transfer brings new challenges for magnetic materials and potential opportunities to open up the market for magnetic materials dedicated to WPT. Although the high-performance power ferrites and nanocrystalline currently used in WPT systems can meet the basic application requirements at this stage, they still have some limitations and large room for performance improvement, as mentioned in the previous section. In response to the current problems of mainstream WPT magnetic materials, the first thing that should be done is to actively respond to the technology needs. An extensive summary of the technical needs of WPT magnetic materials is a prerequisite for finding the right direction for the development of magnetic material technology. Some typical requirements are as follows:

(1) Magnetic Permeability and Resistivity

The magnetic permeability is closely related to the coupling coefficient of the magnetic coupler. The higher the magnetic permeability, the stronger the magnetic coupler’s ability to confine the magnetic field and the larger the coupling coefficient. To a certain extent, a higher magnetic permeability leads to a greater system mutual inductance under the same conditions. This contributes to the power capacity of the WPT system. Moreover, the resistivity determines the parasitic eddy current effect of magnetic materials. For high-performance nanocrystalline as an example, the resistivity and permeability have a non-linear inverse relationship related to the preparation process. Therefore, a compromise between these two parameters needs to be considered in the development of dedicated WPT materials, and different matching combinations of magnetic permeability and resistivity are selected for different application power intervals to form the optimal solution.

(2) Maximum Saturation Magnetic Flux Density

The maximum saturation magnetic flux density of the magnetic materials is related to the theoretical minimum amount of the magnetic core. The greater the transmission power of the magnetic coupler, the greater the magnetic flux density that the magnetic core needs to withstand. In the design of magnetic cores, it is necessary to ensure that the internal flux density of the magnetic material is below the maximum saturation point when the system is operating at the maximum power point and that a margin is allowed. The maximum possible saturation magnetic density is one of the tasks in the development of WPT materials because this property indirectly affects the degree of lightness of the system.

(3) Frequency Applicability Range and Power Loss

The current kilowatt WPT system mainly works in the frequency band in tens to hundreds of kHz. The magnetic loss of the magnetic coupler is mainly composed of hysteresis loss and eddy current loss. Hysteresis loss is the main component of the power loss, which represents the energy consumed during repeated magnetization due to the hysteresis phenomenon. The applicable frequency range of magnetic materials is usually related to the power loss per unit volume of the magnetic core. In general, when the frequency is below the applicable frequency range, the hysteresis loss of the core dominates the total power loss. When the frequency range is higher than the applicable frequency range, the eddy current loss dominates the total power loss. For achieving the high-efficiency characteristics of WPT magnetic materials, it is necessary not only to enhance the applicable frequency range of the materials but also to reduce the power loss of the materials in the operating interval.

(4) Mechanical Characteristics and Costs

In addition to the characteristic parameters mentioned above, there are other factors to consider when developing new materials. The cost of the material determines the scope of its future application and market positioning. The bulk density of the material determines the degree of lightness of the material. The material’s flexibility and deformability facilitate attachment applications in shaped magnetic couplers, where the core is less likely to crack and be damaged by external stress or shock. These practical requirements should be taken into account in the development process of the WPT material.

### 5.2. Rational Utilization of the Existing Products

The development of new materials does not happen overnight. The current high-performance Mn-Zn ferrite, amorphous, and nanocrystalline may not be the best material choices for WPT systems, but they will still be the magnetic material solution in the short-term development trends for a period of time. So, from the perspective of WPT technology developers, what we should do now is look into how to make good use of the available magnetic material products. WPT designers should first consider the aggregated magnetic coupler design ideas in Section 4.1 to play around with the gain of magnetic materials on the main flux of mutual coupling. Furthermore, designers can refer to the refinement crushing and staggered arrangement process mentioned in Section 4.3, the layout direction design of laminated magnetic ribbon materials and the special design of multi-material embedded cores to take full advantage of the existing magnetic materials in the magnetic coupler design.

### 5.3. Promote the Research and Development of Novel Materials Technology

Since there is currently no specialized family of magnetic materials in the field of WPT technology, the development of new specific materials is crucial from a long-term perspective. At this stage, the development of specialized magnetic materials for wireless power transmission can be implemented from a similar approach to designing composite magnetic materials. Firstly, the mapping relationship with the proportional elements of magnetic materials should be established from the perspective of material chemistry according to the WPT requirements. Furthermore, according to the phase analysis, microdomain composition analysis, microstructure analysis, and magnetic property analysis of soft magnetic materials, the design method of composite magnetic materials can be proposed from the perspective of element formulation, preparation and molding process. Eventually, the process flow will be optimized at the level of preparation technology to further improve the thermal and mechanical properties of materials.

With the continuous improvement and popularity of WPT technology, a series of new WPT-specific magnetic materials will soon be available. From the perspective of material companies, the development of new WPT-specific materials can take the lead in capturing market share and enhancing the market influence of the materials, accelerating the maturity of WPT technology. From the perspective of WPT technology R&D companies, the maturity of material production technology is conducive to the popularization and mass production of the material and the reduction of WPT product costs. From the perspective of consumers, the efficiency improvement and reliable operation of the products ensure a good user experience. In conclusion, the new WPT-specific material technology and the advancement in WPT technology are complementary and will synergistically promote the development of related industries in the future.

## 6. Outlook

As of March 2022, retention of China’s new energy vehicles has amounted to 8.915 million, including 7.245 million EVs, accounting for 81.27% of the total new EVs. Compared to the same period last year, the first quarter of 2022 saw a 138.2% increase in newly registered EVs. Up until July 2022, 3.98 million EV charging piles have been set up in China. The electric vehicle industry is growing at a rapid pace internationally, driving a boom in the charging infrastructure market. In this context, wireless charging is gaining widespread attention due to its spark-free, environment-independent and suitability for unmanned operation features. Many famous car companies such as BMW, Infiniti, Volvo, and McLaren have already installed EV-WPT functions in their pilot cars or mass-produced luxury products. Just like when wireless charging technology for smartphones was first introduced, EV-WPT will definitely be accepted and popularized as a convenient charging infrastructure with the global automotive market shifts from traditional fuel vehicles to electric vehicles.

Magnetic materials, as an important part of the magnetic coupler in WPT systems, will be in greater demand, including and not limited to the EV field. It will not be long before the wireless charging technology industry becomes one of the most important markets for magnetic materials and electromagnetic components. A large number of new magnetic materials for various types of wireless charging devices, at all power and frequency levels, will gradually be available, forming a complete line of WPT magnetic materials. The new high-performance magnetic materials will further promote the improvement of high-power density, lightweight, reliability, and electromagnetic compatibility of WPT technology to ensure the efficient, stable, and safe operation of wireless charging devices.

## 7. Conclusions

This paper provides a comprehensive and focused review of magnetic materials and their applications in wireless energy transmission technology, including the latest advances in WPT technology and high-performance magnetic material applications. In addition, this paper provides a comprehensive comparison of WPT projects proposed by representative research institutions and companies and the technical parameters and applicable capabilities of the magnetic material products they use. In addition, key issues and research hotspots concerning the current magnetic materials in WPT technology are pointed out and discussed in detail. Finally, challenges and opportunities associated with magnetic material technology in WPT systems are concluded. With regards to magnetic materials in WPT technology, a fresh perspective on both academia and industry is offered. From a cross-disciplinary point of view, this paper also provides the application requirements and optimization directions of the new WPT magnetic materials for magnetic material companies. It is believed that a large number of novel high-performance magnetic materials will be born in the near future to further promote the performance of typical electric equipment, represented by electric vehicles.

## Figures and Tables

**Table 1:** Ability Comparison of different WPT technologies.

WPT Technologies		Power Range	Frequency Range	Transmission Distance	Transmission Efficiency
Far Field	US-WPT	0.1 mW~10 W	50 kHz~5 MHz	1 mm~5 m	<20%
	MW-WPT	1 mW~3 MW	10 MHz~100 GHz	1 mm~10 m	<30%
	OPWT	1 W~1 MW	100 THz~1 PHz	1 m~10 km	<40%
Near Field	EC-WPT	1 W~3 kW	1 MHz~10 MHz	1 mm~10 cm	70~85%
	MCR-WPT	100 W~1 MW	10 kHz~3 MHz	1 cm~1 m	80~95%

**Table 2:** Comparison of the main advantages and potential shortcomings of different WPT types.

WPT Technologies		Main Advantages	Potential Shortcomings
Far Field	US-WPT	Long effective transmission distance Suitable for mobile applications	High radiation and absorption loss Low efficiency around 10% or less Low power level with a few watts or milliwatts Complex implementation
	MW-WPT	Long effective transmission distance Potential to transfer kW power	High radiation and absorption loss Low transmission efficiency Complex implementation
	OPWT	Long effective transmission distance Potential to transfer kW power	Low transmission efficiency High requirements for transfer directional Environmental permeability dependent
Near Field	EC-WPT	High power transfer up to kW Transfer power through metal Lightweight and low cost	Limited efficiency at a range of about 70%~85% Short transmission distance Low power range due to the plate capacitance High electric field requirement leads to high electronic component stress
	MCR-WPT	High efficiency, possibly higher than 90% High power transfer up to MW Good galvanic isolation Adaptable for various power level ranges	Limited transmission distance of cm level Core loss and eddy current loss are existed

**Table 3:** Results and parameter indicators of research institutions on WPT technology.

Institute	Year	Frequency (kHz)	Gap (cm)	Power Level (kW)	Efficiency
University of Auckland	2011	20	20	2	80%
	2013	20	10~25	7	/
	2015	85	10	1	91.3%
	2017	20	15~20	1	90%
KAIST	2011	100	17	6	72%
	2013	20	12	15	74%
	2014	20	20	27	74%
	2015	20	20	22	91%
Korea Railroad Research Institute	2015	60	5	1000	83%
ORNL	2018	/	15.24	120	85%
	2018	22	12.7	50	85%
University of Michigan	2017	85	15	3	95.5%
Saitama University	2012	50	20	3	90%
HIT	2015	85	15	3	87%
	2017	30	30	85	90%
Chongqing University	2015	20	20	10	82.5%
	2016	40	20	30	90%

**Table 4:** Results and parameter indicators of companies on WPT technology.

Company	Year	Frequency (kHz)	Gap (cm)	Power Level (kW)	Efficiency
Qualcomm Halo	2011	85	22	3.3~20	90%
ZTE New Energy	2014	45	20	30/60	90%
Bombardier	2014	85	6	200	90%
WiTricity	2016	85	25	3.3/7.7/11	91~93%
Zone Charge	2017	85	19	7.7/20/30	90%
Momentum Dynamics	2018	85	30.5	200	95%
INVIS Power	2019	85	14	7.7	90%

**Table 5:** TDK’s PC series Mn-Zn ferrites product parameters.

Parameters	PC40	PC44	PC45	PC46	PC47	PC95	PC90
*μ_i_*(25 °C)	2300	2400	2500	3200	2500	2500	2200
*B_s_*(25 °C)/mT	510	510	530	530	530	530	540
*B_s_*(100 °C)/mT	390	390	420	410	420	420	450
*P_c_*(25 °C, 200 mT, 100 kHz)/mW·cm^−3^	600	600	570	350	600	350	680
*P_c_*(100 °C, 200 mT, 100 kHz)/mW·cm^−3^	410	300	460	660	250	320	320
*T_c_*/°C	215	215	230	230	230	230	250

**Table 6:** Ferroxcube’s 3C series Mn-Zn ferrites product parameters.

Parameters	3C90	3C91	3C92	3C93	3C94	3C95	3C96	3C97
*μ_i_*(25 ℃)	2300	3000	1500	1800	2300	3000	2000	3000
*B_s_*(25 ℃)/mT	430	430	~470	~50	430	530	430	530
*B_s_*(100 ℃)/mT	340	340	400	370	340	410	370	410
*P_c_*(100 ℃, 200 mT,100 kHz)/mW·cm^−3^	~450	<330	<400	~350	<400	<330	<330	<300

**Table 7:** Requirements and recommends of magnetic materials for the transmitter in Qi standard.

Types	Fixed Position Type	Single-Coil Free Position Type	Multi-Coil Free Position Type
Operating frequency	110~205 kHz	140 kHz	105~113 kHz
Material requirements	Low loss and magnetic leakage	High reliability	Low loss and high Bs
Material recommendations	Material 44 (Fair Rite) Material 28 (Steward Inc.) CMG22G (Ceramic Magnetics) DPR-MF3 (Daido Steel) HS13-H (Daido Steel)	Material 78 (Fair Rite) 3C94 (Ferroxcube.) N87 (Epcos AG.) PC44 (TDK Corp.)	Material 78 (Fair Rite) 3C94 (Ferroxcube.) N87 (Epcos AG.) PC44 (TDK Corp.)

**Table 8:** Microstructure and magnetic properties of three typical nanocrystalline materials.

Parameters	Finemet	Nanoperm	Hitperm
Ingredients	Fe_73.5_Si_13.5_B_9_Nb_3_Cu_1_	Fe_90_Zr_7_B_2_Cu_1_	Fe_44_Co_44_Zr_7_B_4_Cu_1_
Grains	α-Fe(Si)	α-Fe	α-FeCo
*D*/nm	10	10	8
*B_s_*/T	1.24	1.65	1.6~2.1
*H_c_*/A·m^−1^	0.53	2.4	10
*μ_i_*/10^3^	100	17	1.8
*λ_s_*/10^−6^	2.1	1	30
*T_c_*/℃	570	770	980

**Table 9:** Characteristic parameters of soft magnetic materials currently available on sale.

Magnetic Material	Material Type	*B_s_* (T)	*H_c_* (A/m)	*μ_r_*	*T_c_* (℃)	*ρ_c_* (μΩ·cm)	*P_c_* (mW/cm^3^)
PC40	Ferrite	0.5	15	2300	200	650 × 10^6^	70 (0.2 T,25 kHz) 420 (0.2 T,100 kHz)
PC90	Ferrite	0.54	13	2200	250	600 × 10^6^	68 (0.2 T,25 kHz) 320 (0.2 T,100 kHz)
PC95	Ferrite	0.53	9.5	3300	215	600 × 10^6^	280 (0.2 T,100 kHz)
HS72	Ferrite	0.41	6.0	7500	130	20 × 10^6^	1500 (0.2 T,100 kHz)
2605SAI (0.025 mm)	Amorphous	1.59	3.2	45,000	392	130	180 (0.4 T,10 kHz)
2714A (0.015 mm)	Amorphous	0.57	0.2	170,000	225	142	91.1 (0.5 T,20 kHz) 303.6 (0.2 T,100 kHz)
FeCuNbSiB (0.018 mm)	Nanocrystalline	1.24	0.53	157,000	843	120	15.4 (0.2 T,100 kHz) 280 (0.2 T,100 kHz)

**Table 10:** A comparison of the characteristics of the EV-WPT system using TDK PC-95 Mn-Zn ferrite core and Hitachi Finemet nanocrystalline materials.

Items	PC95 (Mn-Zn Ferrite)	Finemet (Nanocrystalline)
Magnetic Saturation limit	0.53 T	1.24 T
Mechanical Properties	Brittleness	Flexibility
Additional Eddy Loss	Low	Slight High
Core Loss	280 (0.2 T,100 kHz)	280 (0.2 T,100 kHz)
Core Weight	2.8 kg (5 mm)	2 kg (3 mm)
Cost	≈14 USD/kg	≈40 USD/kg
Coupling Performance	Good	Accepatable (Slight Weak)
Shelding Performance	Good	Accepatable (Slight Weak)

**Table 11:** Limits of typical standards regarding electric field strength, magnetic field strength, and magnetic flux density in the dominant operating range of WPT systems.

Standards	Frequency Range	Electric Field Strength (V/m)	Electric Field Strength (A/m)	Magnetic Flux Density (μT)
ICNIRP 1998	3 kHz~150 kHz	87	5	6.25
	0.15 MHz~1 MHz	87	0.73/*f*	0.92/*f*
	1 MHz~10 MHz	87/*f*^1/2^	0.73/*f*	0.92/*f*
ICNIRP 2010	3 kHz~10 MHz	83	21	27
IEEE C95.1 2005	0.1 MHz~1.34 MHz	614	16.3/*f*	-
	1.34 MHz~3 MHz	823.8/*f*	16.3/*f*	-
	3 MHz~30 MHz	823.8/*f*	16.3/*f*	-

**Table 12:** Applicability and potential shortcomings of shielding methods applied in MCR-WPT.

Categories	Shielding Structure	Reference	Applicability	Potential Shortcomings
Passive shielding	magnetic field shielding	[52,187,251,255]	Medium and low frequency and power level	Insufficient shielding effect
	electric field shielding	[256,257,258]	Medium and low frequency and power level	Additional eddy loss
	Metamaterials shielding	[259,260,261,262,263]	High frequency	Structure and frequency limitations
Reactive resonance shielding	Reactive resonance coils	[241,264,265]	Medium and high frequency	Additional coils and compensators
Active shielding	Active coils	[266,267,268]	Focused areas of MC	Complex structure and large size
Combined shielding	Magnetic core + Aluminum plate	[269,270]	Medium and high frequency and power level	Generally acceptable
	Reactive resonance coils + Active coils	[271,272]	Multi-degree of freedom and open MC	Complex structure and large size

## Data Availability

The data supporting the findings of this study are available by reasonable request to jiantaoz@hit.edu.cn.
